# Deficiency in N-cadherin-Akt3 signaling impairs the blood-brain barrier

**DOI:** 10.1016/j.celrep.2025.115831

**Published:** 2025-06-10

**Authors:** Quinn Lee, Wan Ching Chan, Shuangping Zhao, Harry M. Hailemeskel, Riya Thomas, Mohsin Zafar, Fozia Mir, Peter T. Toth, Kamran Avanaki, Leon M. Tai, Jeffrey Loeb, Yulia A. Komarova

**Affiliations:** 1 Department of Pharmacology and Regenerative Medicine, University of Illinois College of Medicine, Chicago, IL 60612, USA; 2 Department of Anatomy and Cell Biology, University of Illinois College of Medicine, Chicago, IL 60612, USA; 3 Richard and Loan Hill Department of Bioengineering, University of Illinois at Chicago, Chicago, IL 60612, USA; 4 Department of Neurology & Rehabilitation, University of Illinois College of Medicine, Chicago, IL 60612, USA; 5 Fluorescence Imaging Core, University of Illinois Research Resources Center, Chicago, IL 60612, USA; 6 Lead contact

## Abstract

The blood-brain barrier (BBB) restricts the passage of protein-rich fluids through tight junctions (TJs) formed between brain endothelial cells (BECs). BBB restrictiveness diminishes with aging, but the underlying mechanisms remain unclear. BECs establish physical contact with pericytes via N-cadherin homophilic adhesion. In cortex tissue from young and middle-aged patients, the age-related loss of vascular N-cadherin corresponds with the disruption of occludin TJs. Genetic deletion of N-cadherin in ECs impairs occludin TJs, leading to reduced cerebral tissue perfusion and spatial memory deficit. Mechanistically, the assembly of N-cadherin contacts stabilizes occludin TJs via the phosphoinositide 3-kinase p110β-Akt3 circuit, which is disrupted with aging. Furthermore, mutation of occludin Ser471 to Ala destabilizes occludin TJs even in the presence of N-cadherin contacts. These findings highlight a functional role for N-cadherin as a signaling hub that stabilizes occludin at TJs in a phosphorylation-dependent manner, thereby supporting BBB integrity.

## In brief

The key advances in Lee et al. demonstrate the function of N-cadherin in stabilizing occludin TJs in brain microvascular endothelial cells and supporting short-term memory. The study shows that N-cadherin contacts activate the PI3Kβ-Akt3 pathway, leading to the phosphorylation of occludin at S471 and the stabilization of occludin TJs.

## Graphical Abstract

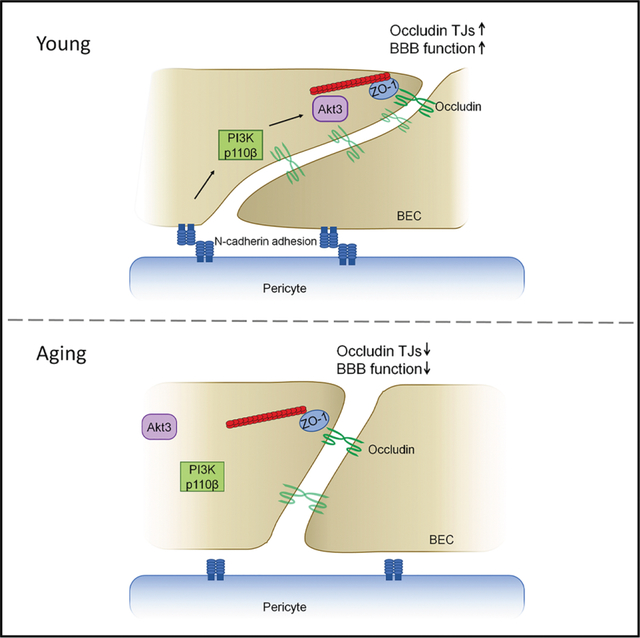

## INTRODUCTION

Aging progressively transforms cognitive function through cell-intrinsic and non-autonomous mechanisms.^1,2^ While neuronal cells exhibit remarkable longevity,^3,4^ the accumulation of neuronal damage,^5^ along with age-related changes in the function of non-neuronal cells,^6^ disrupt various aspects of brain homeostasis contributing to cognitive decline.^5^ A prominent concept underlying brain aging relies on age-related alterations to the brain homeostatic environment.^2^ Recent studies suggest the prevalent contribution of circulating factors in shifting the brain environment toward an inflammatory state during aging.^2^ The studies demonstrate that infusing blood plasma from older animals impairs brain function, whereas plasma from young animals rejuvenates cognition,^2^ underscoring the pivotal role of non-autonomous mechanisms in regulating brain function.

The maintenance of the brain microenvironment is attributed mainly to resident non-neuronal cells, oligodendrocytes, astrocytes, microglia, and brain endothelial cells (BECs) that act in concert to provide continuous support of brain health.^6^ Infectious pathogens and other systemic or local inflammatory responses trigger activation of these non-neuronal cells, altering their normal functions.^2,6^ Among these cell types, particular attention has been given to BECs, as they play a pivotal role in preserving the normal homeostatic environment of the brain.^7^ BECs form the primary barrier of the blood-brain barrier (BBB) through inter-endothelial tight junctions (TJs), which restrict the paracellular transport of molecules.^8^ Together with pericytes, mural cells embedded within the basement membrane,^9^ and astrocytes, glial cells that interact with BECs through their endfeet,^10–12^ BECs constitute the neurovascular unit—a specialized structure responsible for maintaining the BBB as a highly selective interface regulating the exchange of molecules between the bloodstream and the central nervous system.^13^

A distinguishing characteristic of specialized BECs is the presence of TJs, which seal the brain endothelium to prevent neurotoxic proteins and small molecules greater than 400 Da from passing through the paracellular route.^14^ Even in the absence of neurodegenerative pathology, BBB impairment can occur during normal aging.^15–19^ The increase in BBB leakiness to potentially neurotoxic serum proteins, including albumin and autoantibodies, is detectable even in middle-aged individuals and progressively worsens with age.^18,20^ The breakdown of the BBB has long been linked to age-related cognitive decline,^21,22^ supported by evidence of brain vascular hyperpermeability in the hippocampus, a brain region critical for learning and memory.^18^

Brain endothelial TJs are composed of occludin, claudins 1, 3, 5, and 12, and junctional adhesion molecules A–C. These integral membrane proteins form complexes with zonula occludens-1 (ZO-1), a cytoplasmic scaffolding protein that interacts with the actin cytoskeleton to form intercellular contacts.^23^ Interspersed within these zipper-like structures are adherens junctions (AJs), consisting of vascular endothelial (VE)-cadherin forming a complex with α-, β-, and p120 catenin proteins,^24^ which play a secondary role in the brain vasculature. Both occludin and claudin-5 are major components of TJs in the BBB. Claudin-5 is a major TJ protein that forms the backbone of the BBB’s paracellular barrier.^25–27^ Claudin-5 creates TJ strands that seal the inter-endothelial spaces, preventing the passage of solutes and larger molecules between adjacent BECs.^26,28^ It is also involved in regulating the size and charge selectivity of molecules that can cross the BBB.^26^ Genetic deletion of the *Cldn5* gene disrupts the BBB of both newborn and adult mice, resulting in neuroinflammation, seizures, cognitive decline, and lethality.^26,29^

Occludin can synergize with claudins to strengthen TJs and interact with claudins through extracellular loops, contributing to the formation of a restrictive paracellular route.^30,31^ Although occludin alone is not sufficient to form TJs,^32–34^ it stabilizes the junctional complex.^35^ Occludin interacts with intracellular adapters, such as ZO-1, that link TJs to the actin cytoskeleton.^36^ The anchorage of occludin to the actin cytoskeleton is regulated by intracellular signaling. Several lines of evidence indicate that occludin phosphorylation provides the fine control of occludin function at TJs.^37–39^ Phosphorylation of occludin at critical serine and threonine residues is often regarded as a positive regulator of occludin function.^40–42^ Occludin phosphorylation within or proximal to the coiled-coil (CC; amino acids [aa] 413–522) domain can modulate its binding with ZO-1 and paracellular permeability.^43–46^ Here, we provide evidence that the stability of occludin at TJs may be regulated through N-cadherin adhesion-mediated signaling, which is impaired with aging.

Both BECs and pericytes express N-cadherin, a single-pass transmembrane glycoprotein^47^ forming heterotypic adhesion between BECs and pericytes.^48,49^ This adhesion is crucial during development for pericyte attachment to the endothelium forming the capillary wall, with deletion of *Cdh2* in ECs lethal mid-gestation due to vascular deformation.^50^ N-cadherin contacts also support endothelial barrier function in the adult microvasculature.^51^ Our recent work has shown that disruption of N-cadherin contacts, by inducible deletion of *Cdh2* in ECs or pericytes of adult mice, causes increased paracellular permeability in the lung and brain, tissue types enriched with pericytes.^51^ Furthermore, endothelial N-cadherin is lost in mouse brain aging^52^ and therefore may be fundamental to age-related BBB breakdown.

The results presented here show that N-cadherin contacts function as a signaling hub for activating the phosphatidylinositol 3-kinase β (PI3Kβ)-Akt3 circuit for the stabilization of occludin TJs in BECs. Genetic ablation of N-cadherin in ECs causes BBB breakdown, leading to cognitive impairment. We also show that both N-cadherin contacts and occludin TJs are impaired in the brain vessels of middle-aged individuals, further indicating their contributing roles in the mechanisms of physiological aging and cognitive decline.

## RESULTS

### The disruption of N-cadherin and occludin junctions in the aging brain endothelium is a contributing factor to cognitive decline

**Figure 1.**
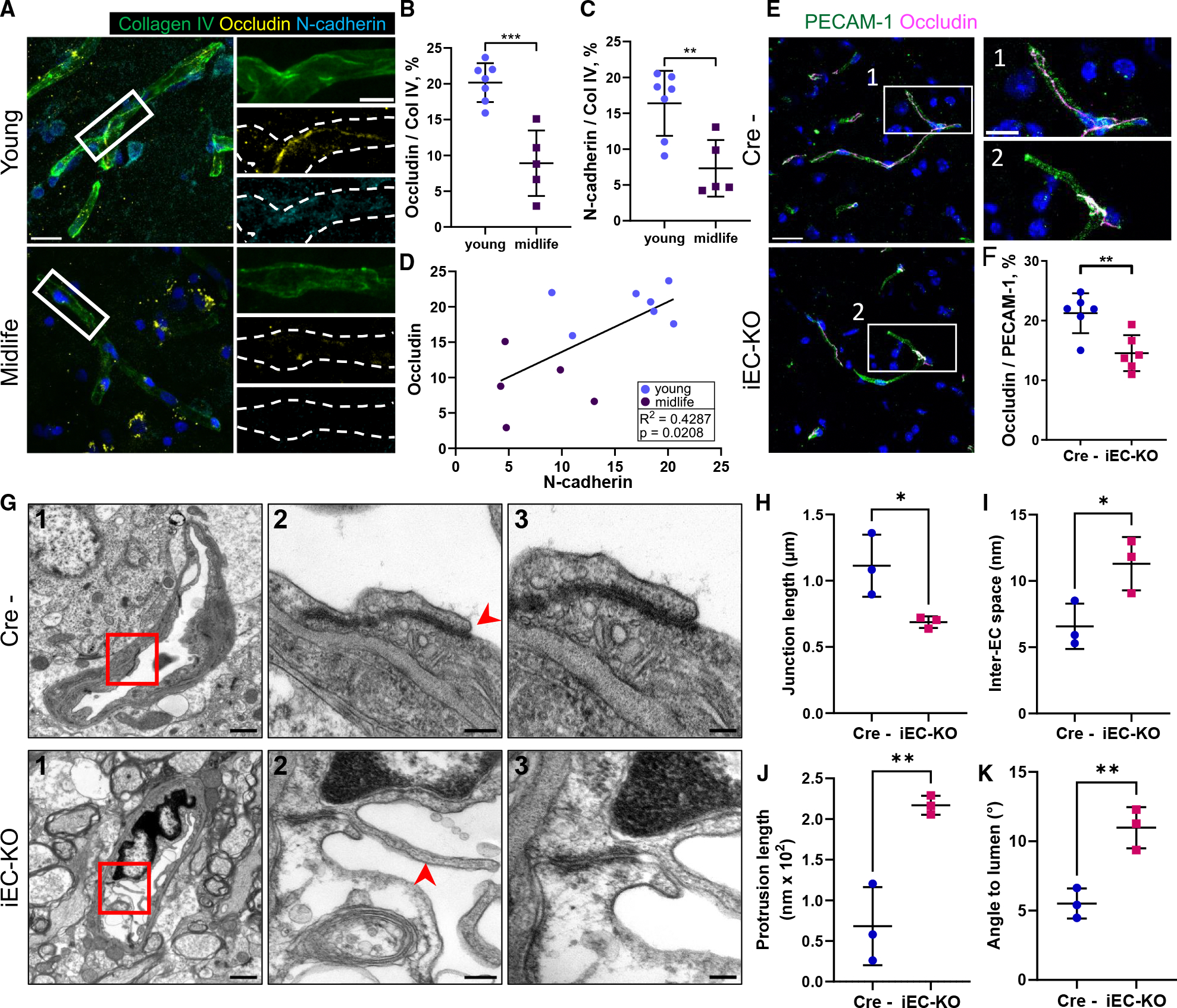
N-cadherin contacts signal the stabilization of occludin TJs (A) Immunofluorescent staining of cortex tissues for occludin (yellow), collagen IV (green), N-cadherin (cyan), and DAPI (blue) from young and middle-aged individuals. Dashed lines outline vessel borders. Scale bars, 20 μm; insert, 10 μm. (B and C) Quantification of N-cadherin (B) and occludin (C) junction areas normalized to collagen IV area in (A). *n* = 5–7 human samples per group; mean ± SD. **p < 0.01; ***p < 0.001 by two-tailed, unpaired t test. (D) Correlation between N-cadherin and occludin junctional areas. Data points from young (blue) and middle-aged (violet) individuals. (E) Representative images of occludin (magenta) and PECAM1 (green) in mouse cortex tissues of N-cadherin fl/fl (Cre^−^, control) and iEC-KO mice. Scale bars, 20 μm; insert, 10 μm. (F) Quantification of occludin junctional area normalized to PECAM1 area in (E). *n* = 6 mice per group; mean ± SD. **p < 0.01 by two-tailed, unpaired t test. (G) Transmission electron microscopy images of brain tissue in N-cadherin fl/fl and iEC-KO mice, including cross-section of vessels (1) and TJs (2, 3) at different magnifications. Red arrowheads point to endothelial protrusions. Scale bars, (1) 1 μm, (2) 0.2 μm, and (3) 0.1 μm. (H–K) Quantification of TJ length (H), inter-endothelial space (I), protrusion length (J), and angle of TJ to vessel lumen (K) using data shown in (G). *n* = 3 mice per group; mean ± SD. *p < 0.05; **p < 0.01 by two-tailed, unpaired t test.

To understand the effects of aging in brain capillaries, we assessed changes in the TJs organization of BECs in human cortex tissue donated from young (18–26 years old) and middle-aged (49–56 years old) individuals (Figures 1A, 1B, and S1A–S1C). We observed a marked loss of occludin at TJs within the capillaries of middle-aged donors (Figures 1A and 1B). These changes correlated with the disassembly of N-cadherin junctions (Figures 1C and 1D) formed between microvascular BECs and pericytes.^48,49^ Junctional localization of claudin-5 or PECAM1, however, was not affected by aging (Figures S1A–S1C).

N-cadherin junctions are prominent regulators of endothelial barrier integrity in organs enriched with pericytes, such as the lung and brain.^51^ To investigate the role of N-cadherin in the organization of occludin TJs, the *Cdh2* gene, which encodes N-cadherin, was inducibly deleted in ECs (*Cdh2*^flox/flox^/end-SCL-Cre-ERT2; hereafter referred to as iEC-KO [knockout]) of young adult mice.^50,51,53^ Similar to the middle-aged group of donors, these young mice showed a significant disruption of occludin TJs compared to control *Cdh2*^flox/flox^ (referred to as Cre^−^) mice (Figures 1E and 1F), whereas claudin-5 was not affected (Figures S1D and S1E). Furthermore, the co-localization of claudin-5 with both occludin and VE-cadherin was markedly reduced in iEC-KO mice (Figures S1F–S1H), consistent with a partial loss of occludin and VE-cadherin junctions. Interestingly, we also observed a reduction in the co-localization of occludin, but not claudin-5, with ZO-1 (Figures S1I–S1L), an adaptor protein that links occludin and claudins to the actin cytoskeleton,^36^ in the brain microvessels of iEC-KO. The co-localization of VE-cadherin with β- and p120-catenin remained unchanged even in the absence of N-cadherin (Figures S2A–S2D), a slight, statistically insignificant increase in co-localization was observed. No change in the protein expression of any junctional proteins was detected in these mice (Figures S2E–S2K), suggesting that the deletion of N-cadherin in BECs may not only alter the assembly of VE-cadherin junctions, as we previously published, ^51^ but also destabilize occludin TJs.

We also observed several ultrastructural abnormalities within TJs of N-cadherin iEC-KO mice (Figures 1G–1K). First, the length of inter-endothelial junctions, characterized by electron-dense material between adjacent BECs, was significantly reduced; however, the paracellular spaces between these junctions were markedly enlarged (Figures 1H and 1I). Second, the outer part of the TJs displayed long protrusions, or “overhangs,” that extended into the vessel lumen (Figure 1J). Third, similar to the phenotype of TJs observed in the brain capillaries of pericyte-depleted mice,^54^ TJs formed a greater angle relative to the lumen (Figure 1K). These data collectively suggest that endothelial N-cadherin plays a critical role in the proper formation of TJs in the brain endothelium.

**Figure 2.**
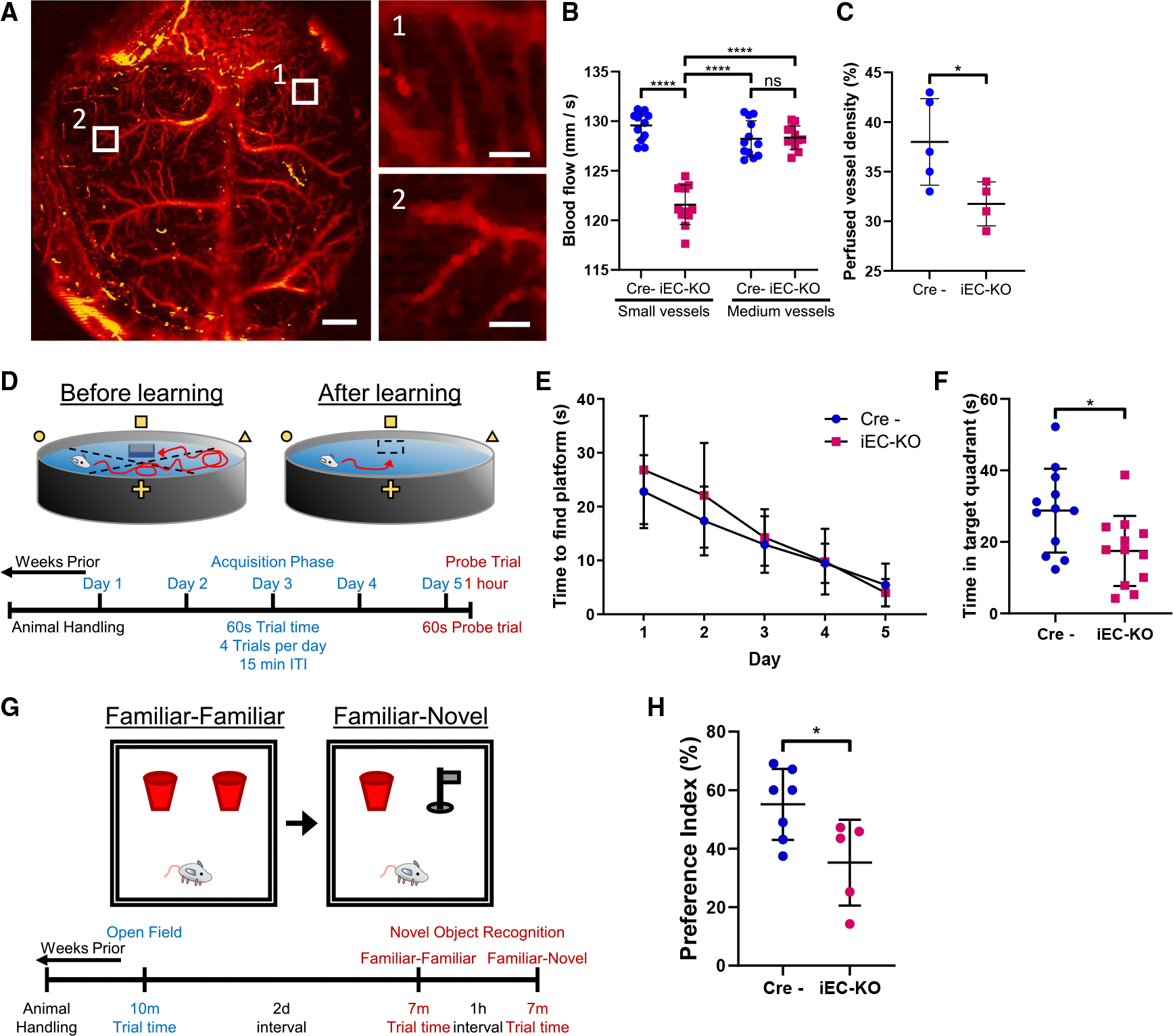
Endothelial N-cadherin supports brain vascular perfusion and spatial memory (A) Representative image of the brain vasculature in mice using photoacoustic microscopy. Scale bars, 1 mm; inset, 0.1 mm. (B) Quantification of blood flow in small- and medium-sized brain vessels of control (Cre^−^) and iEC-KO mice using photoacoustic imaging in (A). *n* = 12 vessels per group in 3 mice; mean ± SD. n.s., not significant; ****p < 0.0001 by ANOVA with Tukey’s post hoc test. (C) Quantification of density of perfused brain vessels of control (Cre^−^) and iEC-KO mice using photoacoustic imaging in (A). *n* = 4–5 regions per group; mean ± SD. *p < 0.05 by two-tailed, unpaired t test. (D) Schematics of Morris water maze test (see STAR Methods) assessing spatial memory in young mice. (E) Time required to find a hidden target platform by control and *Cdh2* iEC-KO mice as a function of training during the 5-day acquisition phase. *n* = 12 mice per group; mean ± SD. (F) Analysis of spatial memory corresponding with data in (D). Time spent in target quadrant by control and *Cdh2* iEC-KO mice during the probe trial of the Morris water maze on day 5. *n* = 12 mice per group; mean ± SD. *p < 0.05 by two-tailed, unpaired t test. (G) Schematics of novel object recognition (NOR) test assessing non-spatial memory. Note that an open field test was conducted prior to NOR to evaluate general locomotor activity, exploratory behavior, and anxiety-like behavior. (H) Novel object preference index of control and *Cdh2* iEC-KO mice during the familiar-novel object trials. *n* = 5–7 mice per group; mean ± SD. *p < 0.05 by two-tailed, unpaired t test.

A deficit in brain capillary perfusion in mice has been observed upon pericyte depletion^55^; thus we next assessed changes in the density of perfused vessels of control *Cdh2*^flox/flox^ and N-cadherin iEC-KO mice using photoacoustic microscopy^56–58^ (Figures 2A–2C). We observed a decrease in blood flow specific to the small-, not medium-sized, vessels of N-cadherin iEC-KO mice (Figure 2B). We also observed a significant decrease in the density of perfused blood vessels (Figure 2C). A similar alteration is observed in aging, as BECs undergo a litany of morphological and functional changes, including impaired cerebral blood flow.^59^

As cognitive decline correlates with increased BBB permeability and aging,^16,18^ and genetic disruption of N-cadherin junctions in mice is also characterized by increased BBB permeability,^51^ we next assessed the cognitive function of N-cadherin iEC-KO mice using the Morris water maze test^60^ (Figures 2D–2F). This is a behavioral test that involves training mice to find a hidden platform in an open swimming area to evaluate spatial learning and memory (Figure 2D). Using this test, we observed that both control and iEC-KO mice demonstrated similar performance during the training phase, indicating no difference in learning ability (Figure 2E). However, N-cadherin iEC-KO mice did exhibit a deficit in short-term spatial memory compared to control littermates during the probe trial (Figure 2F).

To further determine whether N-cadherin deletion in BECs affected general locomotor activity, exploratory behavior, and anxiety-like behavior, we conducted the open field test.^61^ Both groups showed similar total distance traveled, a measure of locomotor activity (Figure S3A). We then assessed recognition memory using the novel object recognition (NOR) test^62^ (Figure 2G). Interestingly, N-cadherin iEC-KO mice demonstrated reduced exploratory behavior, as 7 out of 12 mice spent less than 15 s investigating objects during both the familiarfamiliar and familiar-novel object phases (Figure S3B) and were therefore excluded from further analysis (see STAR Methods). Although total investigation time and preference index were similar between the two cohorts of mice during the familiarfamiliar object phase (Figures S3C and S3D), N-cadherin iEC-KO mice exhibited a significantly lower preference index (Figure 2H) but no difference in total investigation time (Figure S3E) during the familiar-novel object phase. Cumulatively, the results of these behavioral tests indicate that the loss of N-cadherin and, consequently, occludin TJs, may be a contributing factor to memory impairment.

We also determined whether disruption of N-cadherin junctions affected the expression of proteins involved in pre- and post-synaptic neurotransmission. We observed no changes in the expression levels of postsynaptic density protein 95 (*PSD-95*), *N*-methyl-D-aspartate receptor 1, synaptophysin, and vesicular glutamate transporter 1 (data not shown) between the two cohorts of mice. However, the expression levels of synaptic vesicle glycoprotein 2A (SV2A) and glutamic acid decarboxylase 67 (GAD67) were significantly downregulated in the hippocampus of N-cadherin iEC-KO mice (Figures S3F–S3H), corresponding with the diminished memory observed in this group.

### N-cadherin adhesion signals stabilization of occludin TJs

**Figure 3.**
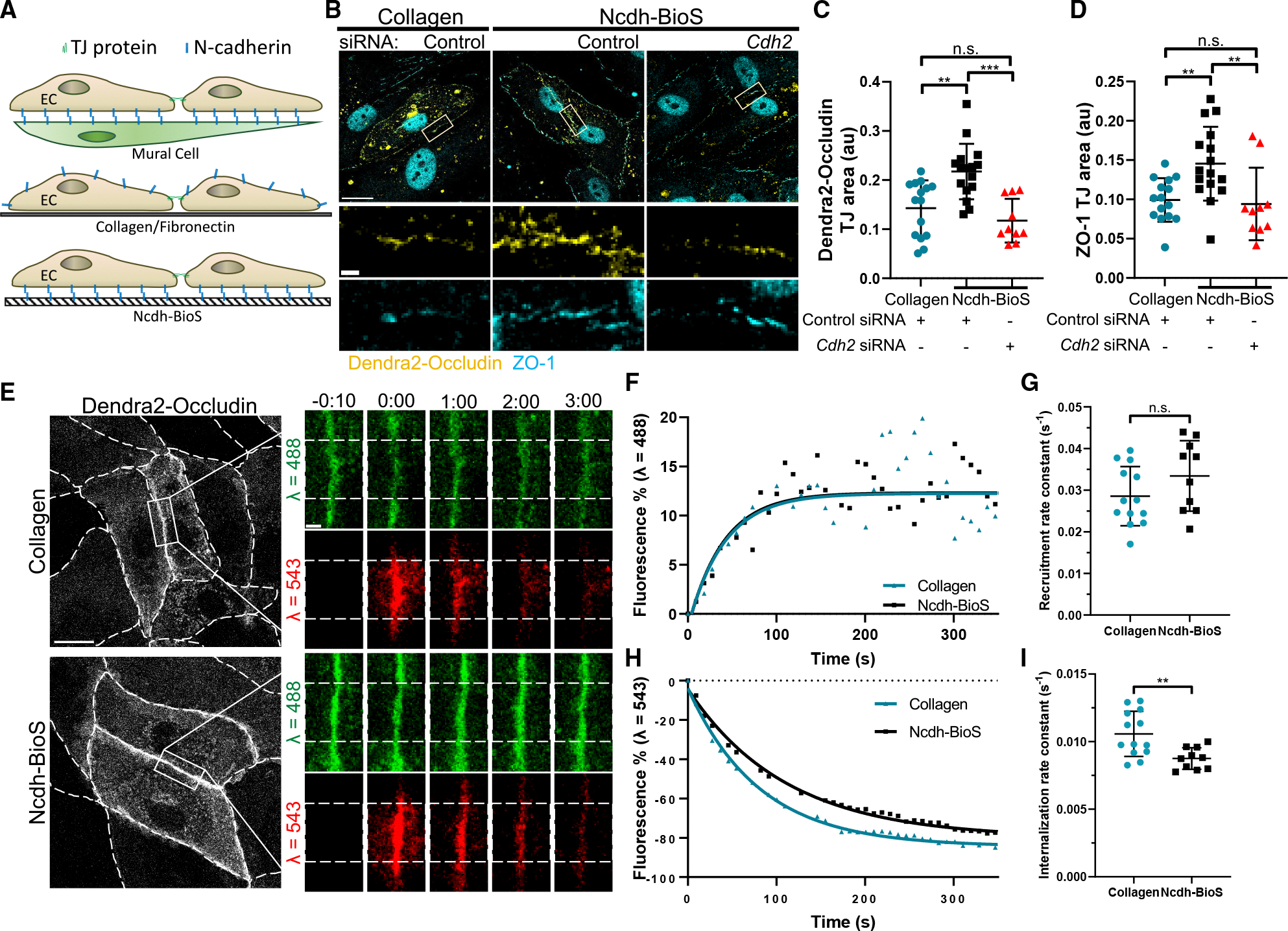
N-cadherin contacts reduce the rate of occludin internalization (A) Schematic representation of N-cadherin biomimetic surfaces (Ncdh-BioS) to induce N-cadherin junctions *in vitro*. (B) Representative images of Dendra2-occludin (yellow) and ZO-1 (cyan) in BECs grown on either collagen or Ncdh-BioS and treated with control (scramble) or *Cdh2* siRNA. Scale bars, 20 μm, inset, 2 μm. (C and D) Quantification of Dendra2-occludin (C) or ZO-1 (D) junctional area in (A). *n* = 10–16 cells per condition; mean ± SD. **p < 0.01; ***p < 0.001 by ANOVA with Tukey’s post hoc test. (E) Time-lapse images of Dendra2-occludin before (green) and after irreversible photoconversion (red) at TJs in BEC monolayers grown on collagen or Ncdh-BioS. Enlarged images on the right: recruitment (green, λ = 488) and internalization (red, λ = 543) of occludin within the irradiation region at TJs (dashed squares) at different times before and after photoconversion at t = 0:00. Dashed lines indicated photoconverted area. Scale bar, 20 μm; inset, 3 μm; time in minutes. (F and H) Representative rates of occludin recruitment (F) and internalization (H) at TJs in (E). (G and I) Recruitment (G) and internalization (I) rate constants (*k*); *n* = 10–13 junctions; mean ± SD. n.s., not significant; **p < 0.01 by two-tailed, unpaired t test.

In most cell culture models, N-cadherin is unable to form junctions as it does *in vivo* between BECs and pericytes. To induce N-cadherin adhesion, we used surface chemistry to covalently attach the extracellular domain of N-cadherin to glass surfaces.^51,63^ By growing BECs on these N-cadherin biomimetic surfaces (Ncdh-BioS), N-cadherin contacts were formed on the ventral cell surface in cultured BEC monolayers^51,63^ (Figure 3A). BECs, which expressed similar levels of Dendra2-tagged occludin across experimental groups, showed significantly greater accumulation of both occludin and ZO-1 at TJs when grown on Ncdh-BioS compared to control conditions (Figures 3B–3D and S4A). Importantly, depletion of N-cadherin reversed these differences (Figures 3B–3D and S4B), indicating that N-cadherin adhesion influenced the recruitment or retention of occludin at TJs.

Thus, to further determine the effects of N-cadherin adhesion-mediated signaling on the kinetics of occludin at TJs, we determined the rates of recruitment and internalization of Dendra2-tagged occludin. The photoconvertible fluorescent probe Dendra2 irreversibly shifts its emission spectrum from the green to red upon irradiation,^64^ enabling the analysis of occludin kinetics within the irradiated region. The concurrent changes in both green and red fluorescence after photoconversion were used to measure the respective recruitment and internalization rates of occludin at TJs. Our data showed that N-cadherin contacts formed in BECs grown on Ncdh-BioS had no effect on the occludin recruitment rate constant (Figures 3E–3G) but significantly slowed the internalization rate (Figures 3E, 3H, and 3I). These data suggest that N-cadherin junctions enhance occludin stability at TJs.

### N-cadherin adhesion activates PI3Kβ signaling to stabilize occludin at TJs

**Figure 4.**
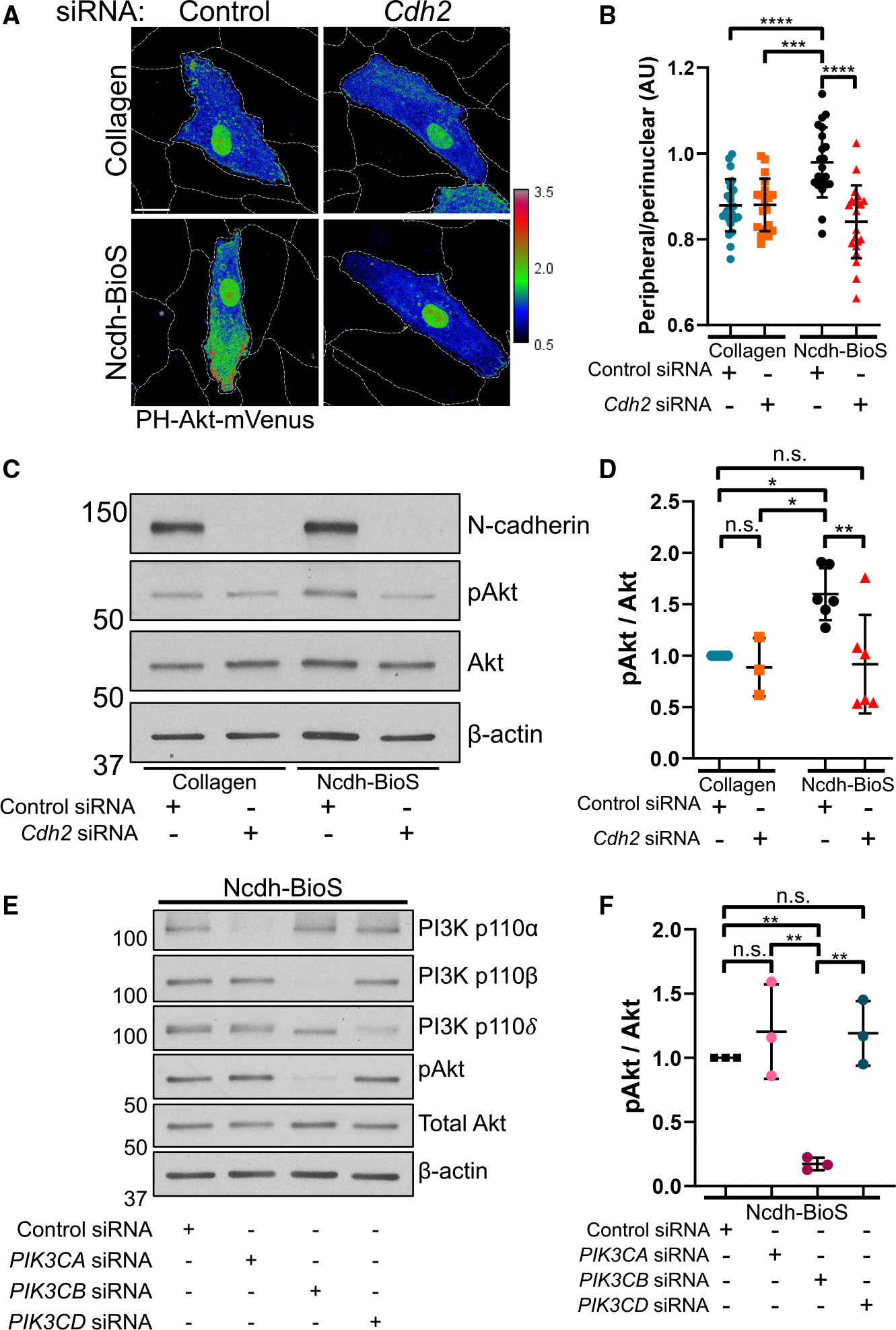
N-cadherin contacts induce Akt signaling through PI3K p110β (A) Projected confocal images of BEC monolayers expressing PH-Akt-mVenus that are grown on either collagen or Ncdh-BioS and treated with control (scramble) or *Cdh2* siRNA. Dashed lines outline cell borders. Scale bar, 20 μm. (B) Ratio of peripheral over perinuclear PH-Akt-mVenus signals in (A). *n* = 19–24 cells per condition; mean ± SD. ***p < 0.001; ****p < 0.0001 by two-way ANOVA with Tukey’s multiple comparisons test. (C) Protein expression of N-cadherin, pAkt, total Akt, and β-actin assessed by western blotting in BECs grown on either collagen or Ncdh-BioS treated with control (scramble) or *Cdh2* siRNA. (D) Quantification of relative pAkt to total Akt ratio in (C). *n* = 3–6 independent experiments; mean ± SD. n.s., not significant; *p < 0.05; **p < 0.01 by two-way ANOVA with Tukey’s multiple comparisons test. (E) Protein expression of PI3K p110α, p110β, p110δ, pAkt, total Akt, and β-actin in BECs grown on Ncdh-BioS and treated with control, *PIK3CA*, *PIK3CB*, or *PIK3CD* siRNA. (F) Quantification of relative Akt phosphorylation expressed as the pAkt-to-total Akt ratio in (E). *n* = 3 experiments; mean ± SD. n.s., not significant; **p < 0.01 by ANOVA with Tukey’s post hoc test.

N-cadherin is known to activate downstream pathways through the recruitment of signaling molecules to the adhesion complex^51,65,66^ or by interacting with receptor tyrosine kinases, for example, fibroblast growth receptor 1 (FGFR1).^67^ Similar to VE-cadherin, which forms a mechanosensitive complex with VE growth factor receptor 2 (VEGFR2),^68^ N-cadherin can engage non-canonical FGFR1 signaling involving the PI3K-Akt pathway.^67,69^ We investigated the N-cadherin-PI3K-Akt signaling circuit in BECs using an mVenus-tagged pleckstrin homology (PH)-Akt sensor, which traces the spatial distribution of Akt at the cell membrane relative to the perinuclear region,^70^ the result of PI(3,4,5)P3 production by PI3K.^71^ We observed a shift in the distribution of PH-Akt sensor toward the cell periphery in BECs grown on Ncdh-BioS compared to control collagen conditions (Figures 4A and 4B). The depletion of N-cadherin, however, prevented the accumulation of PH-Akt at the cell periphery (Figures 4A and 4B), suggesting that N-cadherin adhesion engaged PI3K-Akt signaling.

We also detected biochemically increased phosphorylation of Akt in BECs grown on Ncdh-BioS compared to collagen control, which was reversed by the loss of N-cadherin (Figures 4C and 4D). This biochemical approach supported our observation that N-cadherin activates the PI3K-Akt circuit.

To address the role of N-cadherin-PI3K signaling in stabilizing occludin at TJs, we inhibited the broad spectrum of PI3K with the pan-PI3K inhibitor wortmannin.^72,73^ This treatment accelerated occludin internalization from TJs in cells grown on Ncdh-BioS to the levels observed in cells grown on collagen (Figures S4E–S4I). Furthermore, inhibition of class I PI3K isoforms with Copanlisib (also known as BAY 80–6946)^74^ blocked N-cadherin adhesion signaling as evidenced by reduced phosphorylation of Akt in BECs grown on Ncdh-BioS (Figures S5A and S5B).

To identify the PI3K isoforms activated downstream of N-cadherin adhesion, we depleted catalytic subunits of class I PI3K isoforms using a small interfering RNA (siRNA) approach (Figures 4E and 4F). We found that the depletion of PI3K p110β but not the other catalytic subunits significantly reduced Akt phosphorylation in BECs grown on Ncdh-BioS (Figures 4E and 4F). However, the depletion of PI3K p110β did not affect the Akt protein expression compared to control siRNA-treated BECs (Figure S5C).

**Figure 5.**
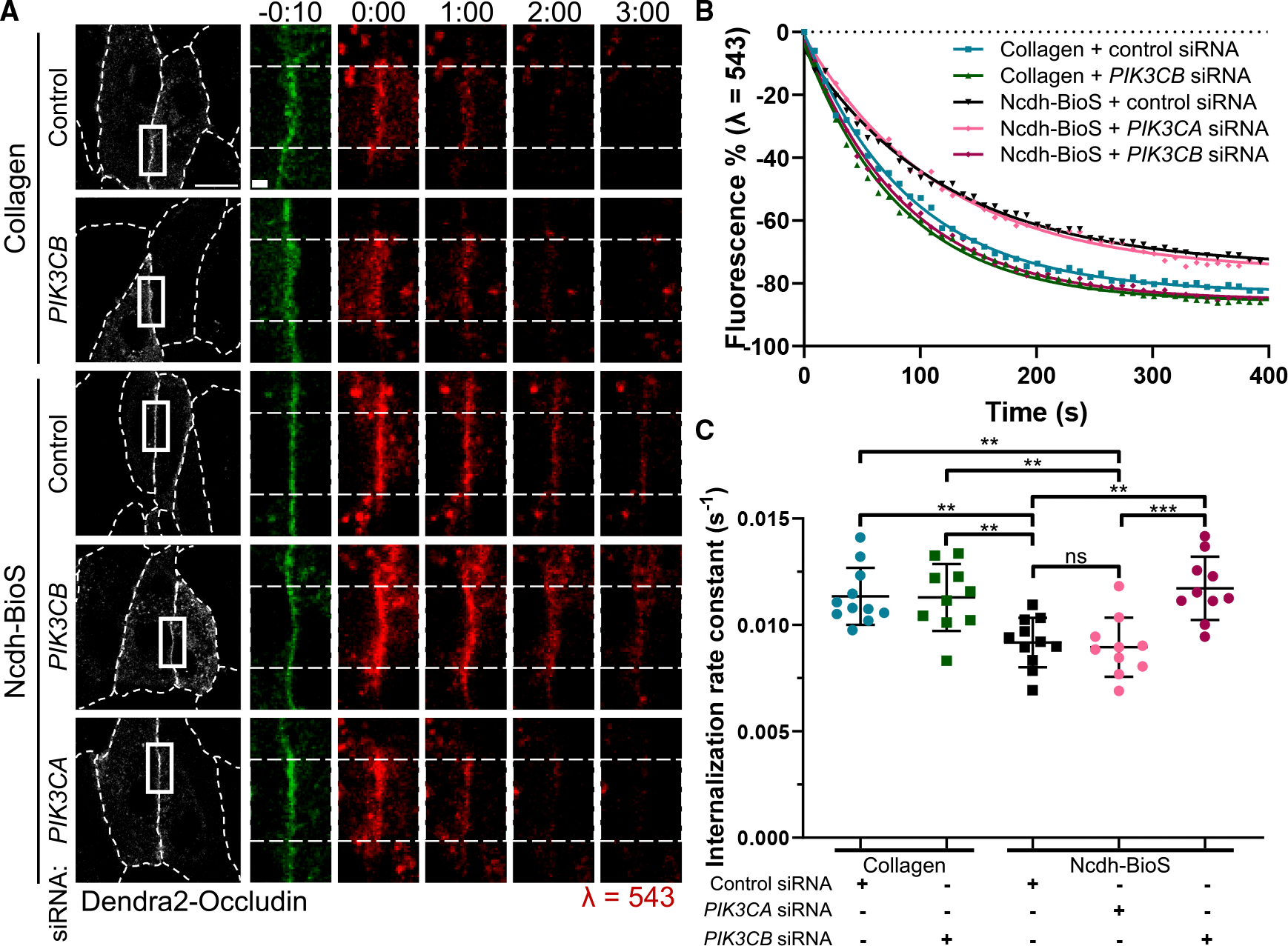
The PI3K p110β is required for stabilization of occludin TJs downstream of N-cadherin contacts (A) Time-lapse images of BEC monolayers expressing Dendra2-occludin that were treated with scramble (control), *PIK3CA*, or *PIK3CB* siRNA and grown on either collagen or Ncdh-BioS. Dashed lines in grayscale images outline cell borders; dashed lines in insert indicate photoconverted TJ area. Scale bars, 20 μm; inset, 2 μm; time in minutes. (B and C) Representative graph of rate of occludin internalization at TJs (B) and internalization rate constant (C). *n* = 10–11 junctions; mean ± SD. **p < 0.01; ***p < 0.001 by one-way ANOVA with Tukey’s post hoc test.

Next, we investigated whether PI3K p110β is required to stabilize occludin at TJs downstream of N-cadherin adhesion. Depletion of PI3K p110β increased the rate of occludin internalization from TJs (Figures 5 and S5D) without affecting its recruitment rate (Figure S5E). However, depletion of the p110α isoform, used as a control, had no effect on occludin kinetics. Furthermore, the changes in internalization rates were observed only in BECs grown on Ncdh-BioS, as PI3K p110β depletion had no effect on the occludin internalization rate in BECs grown on collagen (Figure 5). Overall, these findings suggest that N-cadherin adhesion promotes stabilization of occludin at TJs through PI3K p110β signaling.

### The N-cadherin-Akt3 circuit and occludin S471 phosphorylation stabilize occludin at TJs

**Figure 6.**
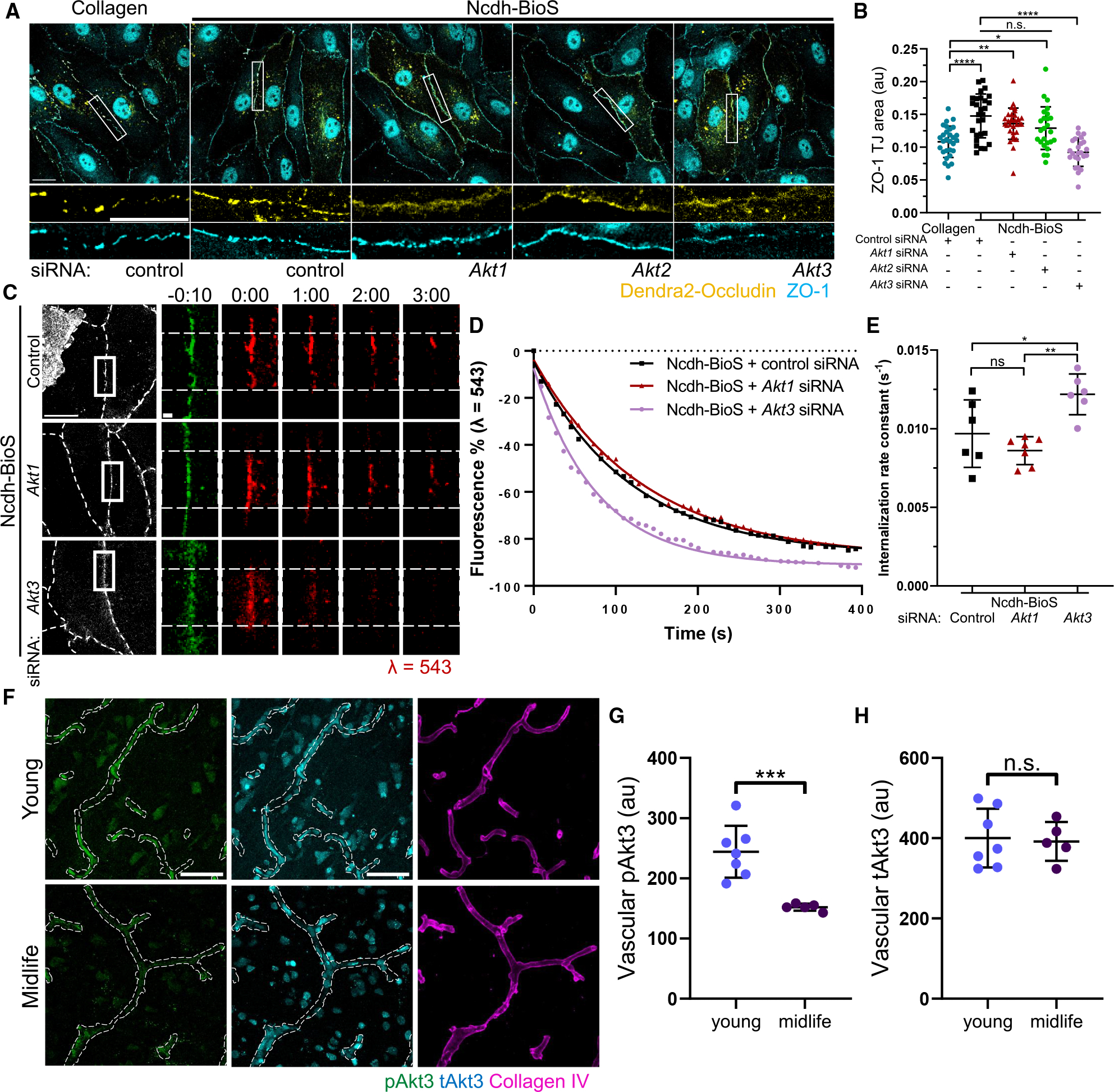
N-cadherin signals the stabilization of occludin TJs via Akt3 (A) Representative images of Dendra2-occludin and ZO-1 (immunofluorescent staining) in BECs treated with control (scramble), *Akt1*, *Akt2*, or *Akt3* siRNA that were grown on collagen or Ncdh-BioS. Scale bars, 20 μm; inset, 20 μm. (B) Quantification of ZO-1 junction area from data in (A). *n* = 24–32 cells per condition; mean ± SD. n.s., not significant; *p < 0.05; **p < 0.01; ****p < 0.0001 by ANOVA with Tukey’s post hoc test. (C) Time-lapse images of Dendra2-occludin in BEC monolayers treated with scramble (control), *Akt1*, or *Akt3* siRNA, and grown on Ncdh-BioS. Dashed lines indicated photoconverted TJ area. Scale bars, 20 μm; inset, 2 μm; time in minutes. (D and E) Representative graph of rate of occludin internalization at TJs (D) and internalization rate constant (E) of occludin from TJs in (C). *n* = 6–7 junctions; mean ± SD. *p < 0.05; **p < 0.01 by ANOVA with Tukey’s post hoc test. (F) Immunofluorescent staining of human cortex tissues from young and middle-aged individuals for pAkt3 (green), total Akt3 (tAkt3; cyan), and collagen IV (magenta). Dashed lines outline vessel borders. Scale bar, 50 μm. (G and H) Quantification of pAkt3 (G) and tAkt3 (H) intensity per vessel area in (F). *n* = 5–7 human samples per group; mean ± SD. n.s., not significant; ***p < 0.001 by two-tailed, unpaired t test.

We next sought to determine an Akt-specific isoform involved in the stabilization of occludin at TJs. We depleted all three Akt isoforms in BECs using an siRNA approach (Figure S6A). As the interaction between occludin and ZO-1 is a key determinant of occludin stability at TJs,^43,75^ in this set of the experiments, we measured the accumulation of ZO-1 at TJs (Figures 6A and 6B). Consistent with the results shown in Figures 3B–3D, the assembly of N-cadherin contacts increased ZO-1 accumulation in BECs treated with control siRNA (Figures 6A and 6B). Depletion of Akt3 but not of Akt1 or −2 reversed these changes to control collagen conditions (Figures 6A and 6B).

We also determined whether Akt3 was required in regulating occludin turnover at TJs. We observed that loss of Akt3 increased the internalization rate of occludin to levels similar to cells grown on collagen (Figures 6C–6E and S6B). However, the depletion of Akt1 had no effect on occludin kinetics, further corroborating the isoform-specific role of Akt signaling in stabilizing occludin at TJs. These data support the notion that N-cadherin contacts stabilize occludin TJs through a PI3K p110β-Akt3 circuit.

We next assessed the levels of Akt3 activity in the microvasculature of the cortex from young and middle-aged donors (Figures 6F–6H). We observed a significant decrease in the phosphorylation of Akt3 in the BECs of middle-aged donors compared to young individuals (Figure 6G), whereas total Akt3 remained unchanged (Figure 6H). This finding aligns with the concept that N-cadherin adhesion stabilizes occludin at TJs via Akt3 signaling and that this regulatory circuit is disrupted during aging.

**Figure 7.**
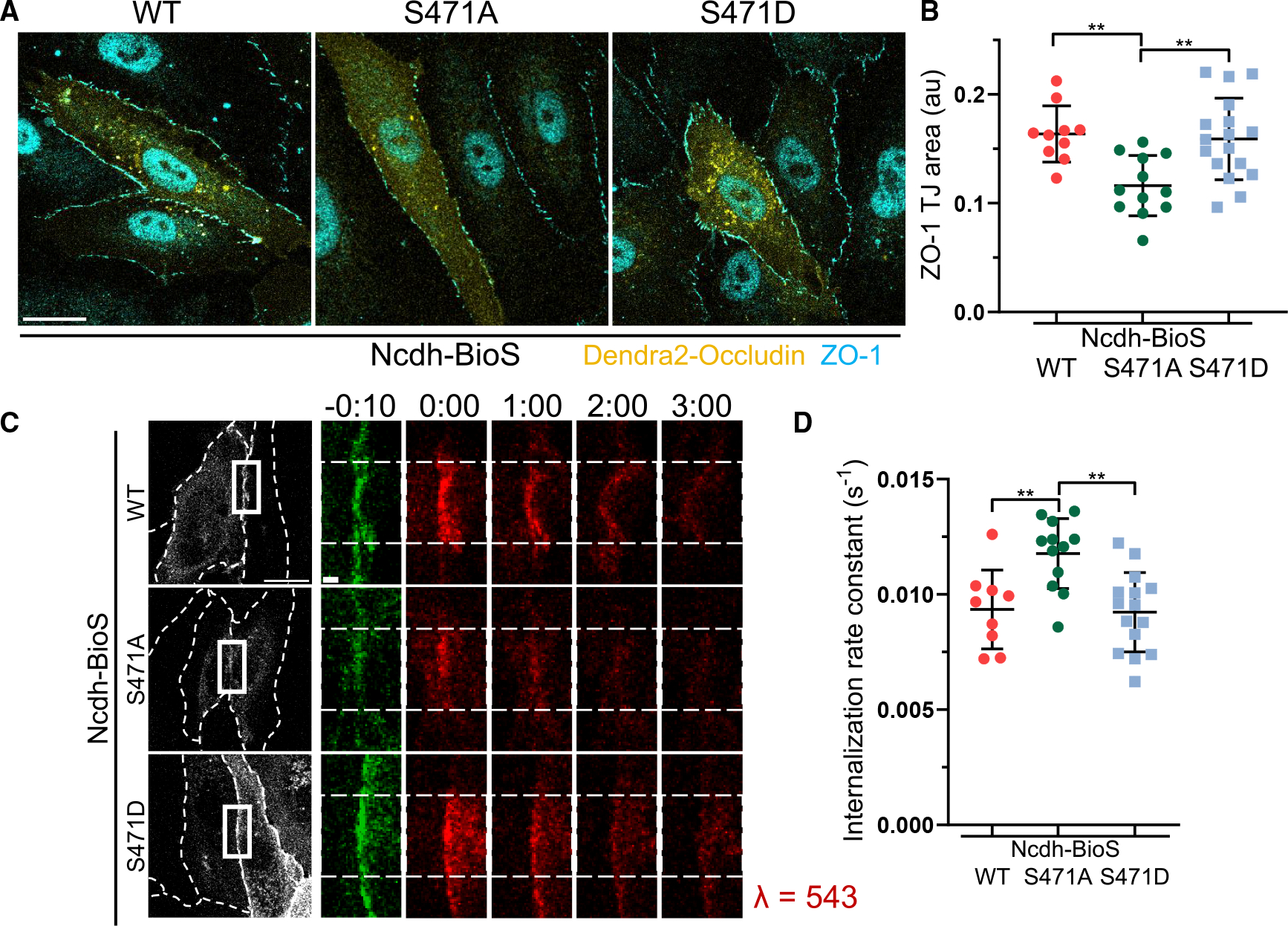
The phosphorylation of S471 stabilizes occludin TJs (A) Representative images of ZO-1 at TJs in BECs expressing Dendra2-tagged wild-type (WT), S471D, or S471A occludin mutants that were grown on Ncdh-BioS. Scale bar, 20 μm. (B) Quantification of ZO-1 junctional area from data in (A). *n* = 10–17 cells per condition; mean ± SD. **p < 0.01; by ANOVA with Tukey’s post hoc test. (C) Time-lapse images of Dendra2-occludin WT, S471D, or S471A mutants in BEC monolayers grown on Ncdh-BioS. Dashed lines indicated photoconverted TJ area. Scale bars, 20 μm; inset, 2 μm; time in minutes. (D) Internalization rate constants of data in (C). *n* = 9–15 junctions; mean ± SD. **p < 0.0 by ANOVA with Tukey’s post hoc test.

Occludin interacts with ZO-1 through its CC domain (aa 413–522)^43,76,77^ in a phosphorylation-dependent manner.^37,43,78,79^ Of several phosphorylation sites within this CC domain, the phosphorylation of S471 promotes the formation of occludin-ZO-1 complexes in epithelial cells.^79^ S471, located within an RXXS/T phospho-motif,^80^ is predicted to be phosphorylated by several kinases, including Akt3.^38,39^ Hence, we investigated whether the phosphorylation state of occludin S471 could similarly influence TJ integrity. Expression of the phosphodeficient S471A occludin mutant significantly reduced the junctional ZO-1 area in BECs grown on Ncdh-BioS as compared to BECs expressing the phosphomimic S471D mutant or wild-type (WT) occludin (Figures 7A and 7B).

Consistent with the role of the occludin-ZO-1 complex in stabilizing occludin at TJs, the phosphodeficient S471A mutant had a significantly greater internalization rate in BECs grown on Ncdh-BioS (Figures 7C and 7D), akin to previous kinetics of cells seeded on collagen-coated surfaces. However, the WT occludin and its phosphomimic S471D mutant had similar internalization rate constants (Figures 7D and S6C). These data cumulatively suggest that the N-cadherin signaling circuit might stabilize occludin at TJs in a phosphorylation-dependent manner.

## DISCUSSION

The results presented here delineate some key mechanisms underlying the breakdown of the BBB during aging. We showed that both N-cadherin contacts, formed between BECs and pericytes, and occludin-containing TJs between BECs are disrupted in middle-aged individuals. These changes in the organization of TJs might contribute to BBB breakdown and cognitive decline associated with aging. Our findings in humans align with data from mice, which reveal an age-related loss of N-cadherin (*Cdh2*) gene expression in BECs.^52^ We also showed that endothelial-specific deletion of the *Cdh2* gene in mice disrupts occludin TJs and significantly alters TJ organization at the ultrastructural level. N-cadherin mutant mice exhibit abnormal organization of inter-endothelial junctions in the capillaries of the mouse cortex, characterized by shorter and more open junctions in the microvasculature compared to control littermates. Similar structural alterations are observed in the cortex of pericytes-depleted mice.^81^ These changes in TJ architecture are associated with increased paracellular permeability,^51^ indicating a potential role for N-cadherin adhesion-mediated signaling in tightening the BBB.

In the model described here, N-cadherin contacts function as a signaling hub to stabilize occludin TJs and support the restrictive BBB. The role of occludin in the organization of TJs is controversial as an occludin null mouse appeared to have intact TJs and normal epithelial barrier function.^82,83^ The lack of a BBB phenotype in these mice might be explained by the presence of an occludin splice variant that was not targeted for deletion.^84^ This truncated variant lacks two exons from the N-terminal side, while still maintaining a transmembrane domain and C-terminal tail, which contains the occludin CC domain for binding to ZO-1.^43^ Hence, this occludin mutant may still cluster and guide TJ organization.

Increasing evidence points to occludin playing a critical role in restricting paracellular permeability *in vitro* ^85–89^ and in injury models *in vivo.*^90–93^ For example, the breakdown of the BBB and blood-retinal barrier following ischemic stroke or challenge with VEGF correlates with increased phosphorylation of occludin at S490 by protein kinase C (PKC)β.^92–94^ This post-transcriptional modification primes occludin for ubiquitin-mediated proteasomal degradation by the E3 ubiquitin ligase, Itch.^92,94^ Breakdown of both barriers is prevented in transgenic mice expressing the phosphodeficient occludin S490A mutant in ECs.^78,93^ An increased rate of occludin degradation is also associated with BBB breakdown under normal physiological conditions, as the depletion of endothelial long noncoding RNA small nuclear RNA host gene (SNHG) 12, which directly interacts with occludin to prevent Itch-mediated degradation, increases basal paracellular permeability without altering claudin-5 expression.^91^ Transgenic mice lacking SNHG12 in ECs exhibit BBB dysfunction and impaired spatial memory.^91^ Hence, our data described here are well aligned with emerging evidence suggesting the critical role of occludin in the maintenance of the BBB under physiological conditions.

Mechanistically, the results of this study show that N-cadherin contacts stabilize occludin at TJs by activating a PI3K p110β-Akt3 circuit to reduce the internalization rate of occludin molecules from TJs. While previous work has established the link between N-cadherin and PI3K in ECs,^69^ the functional contribution of this signaling axis to intracellular processes appears to be wide-reaching and context dependent.^95–100^ PI3K p110α is the most well-established isoform in ECs, with a critical role described in vascular development, angiogenesis,^96,101^ and AJ assembly.^102^ The role of PI3K p110β in the endothelium is diverse, depending on the type of vascular bed.^97,98^ PI3K p110β is demonstrated to promote endothelial survival, and depletion corresponds with loss of anti-aging factor apelin in renal microvessels.^98^ PI3K p110δ and p110γ aid in leukocyte trafficking following inflammatory injury,^103,104^ while PI3K p110γ also promotes repair of the pulmonary endothelium.^105^ Our results in BECs indicate that N-cadherin adhesion induces a PI3K p110β-Akt3 circuit to fortify occludin TJs and the BBB and that the loss of this signaling axis may underlie BBB breakdown in aging.

Akt isoforms differentially regulate BBB permeability in an isoform-specific manner.^106,107^ BECs express all three Akt isoforms (Akt1, Akt2, Akt3), each conferring isoform-specific functions due to their subcellular localization, as well as distinct upstream regulators and downstream targets.^99,100,108–111^ Endothelial Akt1 is continuously suppressed by phosphatase and tensin homolog (PTEN), which dephosphorylates the lipid PI(3,4,5)P3^112^ to maintain a low baseline of Akt1 activity and a low rate of transcytosis across the BBB.^106^ A BEC-specific knockout of *Pten* and the consequent activation of Akt1 promotes E3 ubiquitin ligase NEDD4–2-mediated MFSD2A degradation, thereby increasing the rate of transcytosis and transcellular permeability.^106^ In contrast, Akt2 enhances the BBB through transcriptional upregulation of claudin-5.^107^ Akt2 phosphorylates the transcription factor FOXO1, releasing FOXO1 from a silencer region within the *Cldn5* promoter.^107^ Our data suggest that Akt3 stabilizes occludin TJs by phosphorylating occludin at S471, promoting its interaction with ZO-1.^43,79^ While occludin S471 is predicted to be phosphorylated by Akt3,^38^ our data provide support for the stabilization of occludin TJs in an S471 phosphorylation-dependent manner. As the expression of phosphodefective occludin S471 mutant induces rapid disassembly of TJs, we speculate that Akt3 may be phosphorylating occludin S471 to support BBB integrity.

Occludin phosphorylation, particularly within the C terminus, can modulate its localization, interaction with ZO-1, and paracellular permeability.^37,43–46,113,114^ Our data indicate that the phosphorylation of S471 stabilizes occludin TJs by reducing its internalization rate. Occludin is internalized via clathrin-mediated endocytosis and can be trafficked back to the plasma membrane through the small GTPase Rab13^115^ or set for proteasomal degradation by Itch.^94^ As we observed decreased accumulation of occludin at TJs in BECs deficient of N-cadherin, while the expression level remained the same, we concluded that N-cadherin adhesion-mediated signaling regulates the steady-state remodeling of occludin TJs but not occludin protein degradation. These findings suggest a potential mechanism by which BEC-pericyte interaction promotes the formation of stable occludin TJs, thereby restricting the BBB.

We observed a deficiency in both N-cadherin contacts and occludin TJs, as well as phosphorylated Akt3, in the brain microvessels of middle-aged individuals. These data align with previous work showing an age-associated decrease in N-cadherin transcripts in mouse BECs, suggesting that this may be due to reduced accessibility of the *Cdh2* promoter.^52^ The “closing” of chromatin, often regulated by histone modifications or DNA methylation, suppresses gene transcription. Indeed, renal N-cadherin similarly undergoes gene silencing with age, due to increased hypermethylation of the *Cdh2* promoter proximal to a CpG island.^116^ Our data provide evidence that the age-related loss of N-cadherin contacts may contribute to reduced phosphorylation of Akt3 and disruption of occludin TJs, ultimately leading to alterations in the homeostatic brain environment. We observed positive correlation between the assembly of N-cadherin contacts and stability of occludin TJs both in human and mouse models. Loss of N-cadherin in middle-aged individuals correlated with destabilized occludin TJs. Middle-aged donors also exhibited a loss of Akt3 activity, suggesting an age-related disruption of the N-cadherin-Akt3 circuit. Furthermore, genetic ablation of N-cadherin in BECs of young adult mice not only destabilized occludin TJs in brain microvessels but also led to reduced perfusion of brain tissue and impairment of spatial memory.

In summary, the results presented here demonstrate the critical role of N-cadherin contacts in stabilizing occludin TJs and strengthening the BBB. Engagement of N-cadherin contacts through BEC-pericyte interactions induces a PI3K p110β-Akt3 signaling circuit that reinforces TJ stability. As this signaling mechanism is disrupted with aging, understanding its principles may be fundamental to neurovascular function and brain health, potentially paving the way for therapeutic strategies to mitigate cognitive decline during physiological aging.

### Limitations of the study

This study provides valuable insights into the molecular mechanisms underlying BBB breakdown during aging. Nonetheless, several limitations warrant consideration. Although we employed a combination of *in vitro* EC models and genetically modified mice to modulate N-cadherin function via gain- or loss-of-function approaches, these experimental models may not fully recapitulate the complexity of age-related changes in the neurovascular environment *in vivo*. For instance, while biomimetic surfaces were employed to induce N-cadherin junction assembly *in vitro*, these conditions may not adequately reflect the intricacies of the neurovascular niche. This niche comprises not only ECs but also pericytes, neural stem cells, astrocytes, and glial cells, all of which play critical roles in BBB regulation.^117^ These supporting cell types regulate the stability of TJs, particularly occludin junctions, through both physical interactions with scaffolding proteins such as ZO-1^118^ and the transcriptional regulation of junctional protein expression.^118^ Additionally, the inducible endothelial-specific KO model used to delete the *Cdh2* gene primarily reflects the acute effects of N-cadherin loss on cognitive function. However, aging is characterized by a gradual, progressive decline in N-cadherin expression,^52^ driven by complex and multifactorial processes such as chronic inflammation, metabolic dysregulation, and epigenetic changes, factors that were not addressed in this study.^119^ Moreover, the regional specificity of N-cadherin expression in the brain remains poorly understood. Future studies investigating the distribution, abundance, and transcriptional regulation of N-cadherin across different brain regions during aging could offer deeper insight into the spatial heterogeneity of vascular aging and its contribution to cognitive decline. Additionally, while sex-related differences were not observed in our studies, behavioral studies using only female mice and correlative immunofluorescent analysis using their male littermates limit generalizability. Finally, while this study focuses on the cortex, the lack of observable BBB disruption in other brain regions raises important questions about region-specific vulnerability, which warrants further investigation. Notably, BBB breakdown has been observed to occur as early as middle age in the hippocampus of mice during normal aging. One potential contributor to this regional variability is the differential remodeling and plasticity of TJs^120,121^ or pericyte-BEC interaction,^122^ factors not directly addressed in the present study. Future studies incorporating non-invasive imaging modalities or region-specific molecular profiling of the human brain could help validate and extend these findings, offering a more comprehensive understanding of the spatial heterogeneity of BBB dysfunction in the aging brain.

## RESOURCE AVAILABILITY

### Lead contact

The lead contact for this paper is Dr. Yulia Komarova (ykomarov@uic.edu).

### Materials availability

Any requests for materials regarding this manuscript will be fulfilled by the lead contact.

### Data and code availability

- Any requests for data regarding this manuscript will be fulfilled by the lead contact.
- This manuscript does not contain any original code.
- Any additional information required to reanalyze the data reported in this paper is available from the lead contact upon request.

## STAR★METHODS

### EXPERIMENTAL MODEL AND STUDY PARTICIPANT DETAILS

#### Human samples

Human neocortical tissue section samples were obtained from the University of Illinois NeuroRepository, from patients who underwent surgery for drug-resistant epilepsy.^123^ Patients were enrolled following informed consent as part of a research protocol that was approved by an Institutional Review Board (IRB) at Wayne State University and at the University of Illinois–Chicago (IRB #2015–0457). All data and tissue are stored with de-identified information in the University of Illinois NeuroRepository. Patients underwent a two-stage surgery with long-term subdural electrocorticography (ECoG) and at least 3 days of continuous *in vivo* monitoring to identify the epileptic focus which was then removed during second stage surgery. Only tissue that would otherwise have been discarded was collected for these studies. However, the *en bloc* resections yielded samples ranging from high to no epileptic activity. Once extracted, each tissue sample was divided so that half was immediately frozen on dry ice and stored at −80°C for molecular analysis while the other half was fixed in 4% paraformaldehyde (PFA; Electron Microscopy Sciences, #15712) and later embedded in an optimal cutting temperature compound (OCT; Fischer Scientific, #23–730-571) for histological analysis. Human brain tissue sample from patients aged 18–26 years old were categorized as “young”, while patients aged 49–56 years old were categorized as “middle-aged”. Immunofluorescent staining experiments assessing N-cadherin, occludin, and Akt3, used sample sizes of 7 young (3 female and 4 male) and 5 middle-aged (3 female and 2 male). In the experiment assessing claudin-5 and PECAM-1 utilized a sample size of 4 per condition (young: 2 female and 2 male; middle-aged: 3 female and 1 male).

#### Mice

All animal work performed in this manuscript was approved by the Institutional Animal Care and Use Committee (IACUC) at UIC and conforms to all relevant regulatory standards. Mice were housed with food and water in the University of Illinois Chicago (UIC) animal care facility in a 12 h light and dark cycle. *Cdh2* flox/flox and *Cdh2* iEC-KO mice were generated, authenticated, and maintained as previously described^51^ in which the iEC-KO transgenic mice were generated by crossing *Cdh2* flox/flox mice^124^ with End-Scl-Cre-ERT2 mice.^53^ All mice were of the C57BL/6J genetic background.

To induce genetic deletion of *Cdh2* gene, tamoxifen (Sigma Aldrich, #T5648) was prepared in corn oil (Sigma Aldrich, #C8267) at a concentration of 20 mg/mL and dissolved by shaking at 37°C. Mice at 6–8 weeks of age received intraperitoneal (i.p.) injection with 100 μL tamoxifen once a day for 5 consecutive days. Experiments were conducted at least 2 weeks after Cre induction. Tamoxifen treatment induced endothelial cell-specific deletion in iEC-KO mice. Mice were selected to be similar in age and randomized by sex and transgene presence (control, Cre-negative; experimental group, Cre-positive). In our studies, mice were of an age range from 2 to 4 months. Both groups are treated with tamoxifen as indicated in the protocol. In behavioral studies, only female mice were used as male mice often exhibit anxiety during trail test. For endothelial junction co-localization analyses in mice brain tissue, male littermates to female mice used in open field and novel object recognition behavioral tests were used.

#### Cells

Primary human brain microvascular endothelial cells (BECs) purchased from Cell Systems (#ACBRI 376) were grown in Complete Classic Medium with serum and CultureBoost. Cells were tested for mycoplasma contamination by the vendor and authenticated as positive for endothelial markers CD31 and vWF, as well as positive for Dil-Ac-LDL uptake. BECs were also confirmed to be positive for the endothelial marker VE-cadherin in our hands. Cells (Cell Systems, #4Z0–500) were used at passages 4 to 7 and maintained at 37°C and 5% CO_2_.

### METHOD DETAILS

#### Tissue collection

When collecting tissue, mice are treated with a mixture of xylazine (5 mg/kg) and ketamine (100 mg/kg) for anesthesia and humane euthanasia, before being perfused with phosphate-buffered saline with calcium and magnesium (PBS^+/+^) to remove blood. Brain tissue was harvested and separated into hemispheres. One hemisphere was dissected into cortex, hippocampus, and cerebellum tissue to be flash-frozen in liquid nitrogen for biochemical experiments. The remaining hemisphere was embedded and stored in cassettes with OCT compound for histological experiments. This tissue was cryosectioned into 12 μm saggital slices and adhered to a glass slide. For endothelial junction co-localization analyses, the mice brain tissue was processed by formalin-fixation and paraffin embedding (FFPE).

#### Photoacoustic microscopy

All procedures are conducted following the guidelines outlined in the ‘Guide for the Care and Use of Laboratory Animals’ and were approved by the University of Illinois Animal Care and Use Committee. We used a spiral laser scanning Photoacoustic (PA) microscopy (sLS-PAM) system for imaging the brain vasculature in mice. This microscopy system includes a 532nm pulsed Nd:YAG laser with a pulse repetition rate of 100kHz. The beams are directed onto a 2D galvanometer which are controlled using our pre-established spiral scanning method, the details of which have been described in our prior works.^56,58,125^ The steered beam is focused through an f-theta lens onto the imaging target. The fabricated transducer along with amplifier is positioned at an inclined angle from the imaging plane. The amplified PA signals are digitized at a sampling rate of 100 MS/s. A hollow 10 × 10cm^2^ water tank is used with its base covered by an optically transparent thin film. Mice are placed at the bottom of the water tank with their head pushed against the thin film. Ultrasound gel is applied between the head and thin film to maximize PA signal transmission.

Four regions are selected for calculating perfused vessel density and blood flow in each experimental group. The mice are initially anesthetized with 4% isoflurane gas (1.0 L/min flow rate) via inhalation, and anesthesia is maintained at 1% isoflurane during imaging. Hair from the head is removed using a trimmer to expose the scalp. Subsequently, the entire dorsal scalp is removed, and the periosteum is carefully scraped off from the exposed skull bone using a scalpel blade. Imaging is performed with the skull intact.

A region measuring 10 mm × 10 mm (along the x- and y axes, respectively) is repeatedly imaged over a period of 20 s, with a volumetric imaging speed of 5 Hz. Subsequently, each PAM image is cropped to a size of 1 mm × 1 mm, and an image registration process is applied to all PA images to minimize motion artifacts and correct for scanner nonlinearities. To calculate blood flow, we trace the PA signals inside the capillaries and larger vessels. We estimate the blood flow rate by calculating the changes in flow distance over the duration of image acquisition. To measure vessel density, the amount of blood vessels is calculated within a defined region of the brain. To ensure fairness and accuracy in the comparison, the dimensions of the region were kept constant for all animals in both experimental groups.

#### Morris water maze behavioral assessment

The behavioral test Morris water maze was conducted as previously described.^126,127^ A 1.2 m diameter × 0.5 m tall round pool was filled with water that was made opaque using non-toxic tempera paint to hide a single escape platform. This pool was virtually divided into four equally sized quadrants that were marked with distinct visual cues. Mice behavior was tracked and analyzed using the ANY-maze software (Stoelting Co.). On the first day of the acquisition phase, mice were individually placed on the hidden platform for 15 s.

Afterward, mice were trained to find the location of the hidden platform over the course of the five-day acquisition period, with 60 s trials four trials per day and a 15-min inter-trial interval between trials. While the starting point of each trial was from a different quadrant, the location of the hidden platform did not change. During this acquisition phase, mice were measured on the time taken to find the hidden platform as a measure of spatial learning. Mice were assessed of spatial short term memory during the probe trial, where 1 h following the last acquisition trial mice were tested in a single trial after the hidden platform was removed. Mice were measured on the elapsed time in the platform’s quadrant.

#### Open field behavioral assessment

A 46 cm × 46 cm × 40 cm tall square, white arena was used for open field test. Mice were individually placed in the arena and were measured on the total distance traveled for 10 min using the ANY-maze software.

#### Novel object recognition behavioral assessment

Novel object recognition test was performed 48 h after open field test using a 46 cm × 46 cm × 40 cm tall square, white arena. Familiar (red plastic shot glasses) and novel (gray plastic flags) objects were used for investigation. During the familiar-familiar phase, mice were placed in the arena containing two identical familiar objects for 7 min. The familiar-novel phase was conducted 1 h afterward, in which mice were placed in the arena containing one familiar object and one novel object for 7 min. Using the ANY-maze software, mice were assessed on the total investigation time and preference index (the percentage of time spent investigating the novel object relative to the total investigation time). One mouse was excluded due to excessive jumping. Mice with a total investigation time ≤15 s in either the familiar-familiar or familiar-novel phase were excluded (Figure S2B).

#### Immunofluorescent staining

Fresh frozen mice tissue sections were fixed with 4% PFA in PBS^+/+^ (Corning, #21–030-CM) for 10 min. For staining experiments detecting tight junction proteins, samples were instead fixed in ice-cold ethanol for 20 min. After washing samples with PBS^+/+^, tissue sections were permeabilized with 0.25% Triton X-(Fisher BioReagents, #BP151) and blocked with 5% bovine serum albumin (BSA; Sigma Aldrich, #A7906) for 2 h to reduce non-specific binding. Samples were incubated in primary antibodies for Occludin (1:100, #71–1500, Invitrogen), PECAM1 (1:10, #550274, BD Biosciences), and Claudin-5 (1:100, #34–1600, Invitrogen) overnight at 4°C, followed by secondary antibodies (1:200) for 2 h at room temperature. Immunofluorescent staining of cells with ZO-1 (1:100, #33–9100, Invitrogen) was performed as previously described, except cells were permeabilized with 0.1% Triton X-solution and blocked with 3% BSA.

For endothelial junction co-localization analyses, FFPE mice brain tissue were deparaffinized and rehydrated using sequential 3 min washes of 100% xylene, 50% xylene:ethanol, 100% ethanol, 95% ethanol, 70% ethanol, 50% ethanol, and water. After antigen retrieval in a solution of 10 mM Tris, 1 mM EDTA, 0.05% Tween 20 (pH 9.0) for 20 min at 95°C, tissue staining proceeded as previously described from the PBS washing step. After samples were stained and imaged for Occludin (1:100, #71–1500, Invitrogen) and Collagen IV (1:50, #AB769, Sigma-Aldrich), samples were stripped for re-staining experiments as previously reported.^128^ Briefly, samples were incubated in a 25 mM glycine 1% SDS antibody elution buffer (pH 2.0) for 1 h at 50°C. After extensive washing with PBS^+/+^, samples underwent the immunofluorescent staining procedure again from the blocking step and stained for Claudin-5 (1:100, #34–1600, Invitrogen), VE-cadherin (1:100, #555289, BD Biosciences), and Collagen IV. In addition, Claudin-5 (1:100, #34–1600, Invitrogen), VE-cadherin (1:100, #555289, BD Pharmingen), β-catenin (1:40, #SAB4300470, Sigma-Aldrich), p120 catenin (1:25, #sc-23873, Santa Cruz; 1:100 secondary antibody), ZO-1 (1:100, #33–9100, Invitrogen), and Collagen IV (1:40, #134001, Bio-Rad) was stained and imaged as described above without the re-staining procedure.

Human brain tissue samples underwent antigen retrieval by incubating in 10 mM sodium citrate buffer (pH 6.0) for 20 min at 95°C. After permeabilization with 0.1% Triton X-solution and blocking with 3% BSA, samples were incubated in primary antibodies for Collagen IV (1:50, #AB769, Sigma-Aldrich), Occludin (1:100, #33–1500, Invitrogen), N-cadherin (1:100, #ab18203, Abcam), phospho-Akt3 (1:100, #PA5–12898, Invitrogen), PECAM1 (1:100, # ab9498, Abcam), and Claudin-5 (1:100, #34–1600, Invitrogen) and secondary antibodies as previously described. After samples were stained and imaged for phospho-Akt3, samples underwent re-staining for Akt3 (1:100, #8018, Cell Signaling Technology) and Collagen IV as previously described.

All samples were mounted with Fluoromount-G (SouthernBiotech, #0100–01) and imaged using a Zeiss LSM 880 confocal microscopy with an oil-immersion 40X EC Plan-Neofluar or 63X/1.4 NA Plan-Apochromat objective.

#### Transmission electron microscopy of brain tissue

Mice were perfused with Hank’s Balanced Salt Solution with calcium and magnesium (HBSS^+/+^) to remove blood and then by a fixative solution consisting of 2.5% glutaraldehyde, 4% PFA, 0.1 M HEPES, and 2 mM calcium chloride (Electron Microscopy Sciences [EMS], #16220, #15712, #12340; Gibco, #15630080). Cortex tissue was dissected, cut into 1 mm cubic pieces, and then incubated in fixative solution overnight at 4°C. After washing with 0.1 M sodium cacodylate buffer (EMS, #11652), tissue samples were post-fixed in Palade’s osmium consisting of 0.04 M acetate buffer, 1% osmium tetraoxide, and 0.02 N hydrochloric acid (EMS, #11482–56, #19150, #16760) while protected from light. Afterward, tissue samples were stained in Kellenberger’s buffer containing 0.04 M acetate buffer, 0.028 N hydrochloric acid, and 5 mg/mL uranyl magnesium acetate (EMS, #22500) overnight at 4°C while protected from light. Solutions with increasing concentrations of ethanol (50%, 70%, 95%, and 100% ethanol; EMS, #15055) were used to dehydrate the tissue sample. Afterward, the samples were placed in the transitional solvent propylene oxide (EMS, #20412), and subsequently incubated in an equal mixture of propylene oxide and Embed-812/DER 73 embedding solution (EMS, #14130) overnight. Individual tissue pieces were then embedded and cured in flat molds for 3 days at 60°C. The embedded tissue blocks were sectioned, tissue grids post-stained, and brain vessels were imaged using a FEI Spirit G2 transmission electron microscope.

#### Plasmids and transfection

The photoconvertible fluorescent tool, Dendra2, was attached to the N terminus of human occludin protein. The Dendra2-occludin construct was generated from human mEmerald-Occludin-C14 (a gift from Dr. Michael Davidson; Florida State University) and pcDNA3-VE-cadherin-Dendra2,^129^ in the CMV vector and pShuttle-CMV vector using the services of Genscript USA Inc. The Dendra2-occludin adenovirus was generated using the pShuttle-CMV vector at the Cardiovascular Services of the University of California Davis. GFP-occludin S471D and GFP-occludin S471A were gifts from Dr. David Antonetti (University of Michigan).^79^ Dendra2-occludin S471D and Dendra2-occludin S471A was generated by mutagenesis using CMV Dendra2-occludin using the services of Genscript USA Inc. PH-Akt-mVenus was a gift from Dr. Andrei Karginov’s lab.^70^ This construct consists of the PH domain of Akt, which is redistributed to the plasma membrane/cell periphery relative to the cytoplasmic/cell perinuclear region during PIP3 production by PI3K,^71^ serving as a sensor for PI3K activity.

Cells were transfected with PH-Akt-mVenus or phosphomutant Dendra2-occludin by electroporation using an Amaxa Nucleofector Kit for Primary Endothelial Cells (#VPI-1001) according to the manufacturer’s protocol. Experiments were conducted approximately 24 h after transfection.

#### Generation of N-cadherin biomimetic surfaces

N-cadherin biomimetic surfaces were created similarly as previously described.^51,63^ High-precision #1.5 glass coverslips (ZEISS, #474030–9000-000) or glass petri dishes were cleaned and activated with 100% ethanol, 100% acetone, 12 N hydrochloric acid, and 5 N sodium hydroxide (Fisher Chemical, #A18–4, #A144S-500, #SS256–500) for 30 min each, with ddH2O washes in between. After the glass was completely dried, it was functionalized by incubating in dry toluene vapor with 2% (3-Glycidyloxypropyl)trimethoxysilane (Glymo; Sigma Aldrich, #440167) overnight, washed, and incubated at 60°C with 0.01 M sodium carbonate (Fisher Chemical, #S263–500) with 2% *N*-(5-Amino-1-carboxypentyl)iminodiacetic acid (AB-NTA free acid; Dojindo Molecular Technologies, #A459–10) overnight. This was followed by an incubation of 10 mM nickel (II) chloride and 5 mM glycine (pH 8.0) solution for 2 h. To immobilize N-cadherin fragments, His-tagged extracellular domain of N-cadherin (Sino Biological, #11039-H08H) with fibronectin and collagen (MilliporeSigma, #C8919, #F1141) were coated on the Ni-NTA surfaces. Ni-NTA surfaces incubated with fibronectin and collagen, but not N-cadherin, served as the control. The protein was covalently conjugated by a solution of 50 mM (1-ethyl-3-(3-dimethylaminopropyl)carbodiimide hydrochloride) (EDC; ThermoFischer, #22980), 100 mM *N*-Hydroxysuccinimide (NHS; Sigma Aldrich, #130672), and 20 mM (N-2-hydroxyethylpiperazine-N-2-ethane sulfonic acid) (HEPES) (pH = 7.0). The glass was then washed with 1 M imidazole (Sigma Aldrich, #I5513), 10 mM Ethylenediaminetetraacetic acid (EDTA; Research Products International, #E57020–500), and 100 mM sodium chloride in 20 mM HEPES (pH = 7.5) to remove noncovalently linked protein.

#### siRNA and inhibitor treatment

Cells at approximately 70% confluence were treated with control scramble or target protein siRNAs (in the key resources table) with the GeneSilencer siRNA Transfection Reagent kit (Genlantis, #T500750) according to the manufacturer’s protocol. Experiments were performed 72 h after siRNA treatment. Knockdown of protein of interest was confirmed by western blot analysis.

For studies using inhibitor treatment, HBEC confluent monolayers were incubated with 100 nM Wortmannin (TOCRIS Bioscience, #19545–26-7) for 1 h or 100 nM Copanlisib (Selleckchem, #S2802) overnight.

#### Western blotting

For *in vitro* experiments, cells were lysed and collected using radioimmunoprecipitation assay (RIPA; Sigma Aldrich, #R0278) buffer containing protease and phosphatase inhibitors (Sigma Aldrich, #P8340, #P5726, #P0044). For mice samples, dissected brain tissue was collected and homogenized using 1% sodium dodecyl sulfate (SDS) containing protease and phosphatase inhibitors (Fischer Scientific, #S299–100; MilliporeSigma, #450243–10G; Sigma Aldrich, #P8340, #P5726, #P0044).

The Pierce Bicinchoninic acid (BCA) protein assay kit (Thermo Scientific, #PI23225) was used in accordance to the manufacturer’s instructions to measure total protein concentration. Samples were normalized and prepared with Laemmli sample buffer (Bio-Rad Laboratories, #1610416), boiled, underwent gel electrophoresis, and transferred to nitrocellulose membrane. Membranes were blocked with 5% BSA for 2 h to reduce non-specific binding. After incubating in the indicated primary antibody overnight at 4°C for N-cadherin (#ab18203, Abcam), pAkt (#9271, Cell Signaling Technology), Akt (#9272, Cell Signaling Technology), PI3K p110α (#4249, Cell Signaling Technology), PI3K p110β (#3011, Cell Signaling Technology), PI3K p110δ (#34050, Cell Signaling Technology), ZO-1 (#33–9100, Invitrogen), VE-cadherin (#sc-6458, Santa Cruz), β-catenin (#SAB4300470, Sigma-Aldrich), p120 catenin (#sc-1101, Santa Cruz), Occludin (#ab167161, Abcam), claudin-5 (#34–1600, Invitrogen), SV2A (#ab32942, Abcam), GAD67 (#ab26116, Abcam), Akt1 (#2938, Cell Signaling Technology), Akt2 (#3063, Cell Signaling Technology), Akt3 (#8018, Cell Signaling Technology), and β-actin (#sc-47778, Santa Cruz) and subsequently horseradish peroxidase (HRP)-conjugated secondary antibodies for 2 h, membranes were developed and samples probed of indicated proteins by enhanced chemiluminescent (ECL) substrate (Thermo Scientific, #PI32106). Quantification of band density was performed using gel analysis procedure by ImageJ (FIJI) software.

In experiments detecting isoform-specific depletion, PI3K proteins were detected in the order of PI3Kα → PI3Kβ → PI3Kδ and Akt proteins were detected in the order of Akt2 → Akt3 → Akt1.

#### Analysis of junction area

To analyze the area of occludin, claudin-5, or N-cadherin adhesion area in tissue, z stack images were projected for each color channel and then thresholded to generate a binary mask. The overlapping area of occludin, claudin-5, or N-cadherin was calculated utilizing a “bitwise AND” operator with the PECAM1 or collagen IV vessel markers as the second operand. The positive pixels of the corresponding result of occludin, claudin-5, and N-cadherin adhesion area were normalized to PECAM1 or collagen IV.

The ZO-1 and occludin adhesion area in cells was calculated after generating a binary mask by thresholding a z-projected image. The area of the ZO-1 and occludin junction was quantified as positive pixels within an 8–15 pixel wide band of the cell perimeter.

#### Analysis of co-localization of junctional proteins

Image stack pairs obtained from the same region of interest but stained and restained with different antibody regimens were coaligned based on the collagen channel staining with the help of the “Correct 3D Drift” ImageJ plugin. 3D colocalization was calculated by the ImageJ JACoP plugin (https://doi.org/10.1111/j.1365-2818.2006.01706.x) and expressed as Mander’s M1 and M2. Since Mander’s computation is sensitive to noise, image stacks were medium filtered and thresholded to background values before Mander’s computation. Co-localization of VE-cadherin, occludin, and claudin-5 with junction-associated catenin proteins and ZO-1 was assessed on different sections and different sets of vessels. Because catenin proteins are expressed in various cell types in the brain, we removed signals outside the collagen IV-positive area before computing Mander’s coefficient.

#### Assessment of brain endothelial ultrastructure

The length and inter-endothelial space of electron-dense brain endothelial junctions, and the length of the endothelial protrusions after the junctions were quantified using the line segmentation tool in ImageJ. The angle of the junction was measured respective to the vessel lumen. The vessel diameter was calculated as median of the minimum and maximum Feret’s diameter using the outline of the vessel perimeter. Vessels with a diameter less than 8 μm was defined as a capillary,^130–132^ and vessels with larger diameters were excluded from analysis.

#### Measurement of Dendra2-occludin kinetics at TJs

Brain endothelial cells expressing Dendra2-occludin were lived imaging at 37°C and 5% CO2 in the 488 nm and 543 nm channels using a Zeiss LSM 710 confocal microscope equipped with a 63x/1.4 NA Plan-Apochromat objective and binary gallium arsenide phosphide (GaAsp) detectors. A region of interest (ROI) was excited by a 405 nm laser at 10–12% laser power for irreversible Dendra2 photoconversion from green to red.

To measure the rate of occludin recruitment to the tight junction, the change in fluorescence intensity (λ = 488 nm) in the ROI was quantified as the percent difference of each time point to the total fluorescent intensity measured before photoconversion. This was fitted into an one-phase association non-linear curve to calculate the recruitment kinetics, with the formula: Y = Y_0_ + (P-Y_0_)*(1-e^−*k*x^), where P is the plateau of Y and *k* is the rate constant. To quantify the rate of occludin internalization from the TJ, the fluorescence intensity (λ = 543 nm) change in the ROI was quantified as the percent difference of the timepoints to the total fluorescent intensity after photoconversion, with the internalization rate constant *k* was measured using one phase decay non-linear fit, defined as: Y = (Y_0_ – P)*e^−k^*^X^ + P.

#### Analysis of PI3K activity using PH-Akt sensor

To assess PI3K activity, live brain endothelial cells expressing PH-Akt-mVenus sensor were imaged in 37°C and 5% CO2 conditions in the 514 nm channel using a Zeiss LSM 710 confocal microscope equipped with a 63x/1.4 NA Plan-Apochromat objective. Differential interference contrast (DIC) images were captured to visualize the endothelial monolayer. Quantification of z-projected images was conducted similar to what was performed.^70^ The 12 μm area surrounding the cell nuclei was defined as the perinuclear region, and the area within the cell border excluding the perinuclear region was defined as the peripheral region. The ratio of the average intensity of the cell peripheral to perinuclear region was calculated.

#### Analysis of phospho-Akt3 and total Akt3

To quantify the signal of phospho-Akt3 and total Akt3 in human brain tissue samples, z-projected images were created for pAkt3, tAkt3, and collagen IV channels. After thresholding, the total area of collagen IV was measured using a binary mask. The mean intensity of an 82×90 pixel ROI was used for background subtraction of the pAkt3 and tAkt3 channels. Afterward, the signal of pAkt3 and tAk3 outside of brain vessels were excluded using the collagen IV binary mask, and the integrated intensity of pAkt3 and tAkt3 was normalized to the collagen IV area.

### QUANTIFICATION AND STATISTICAL ANALYSIS

GraphPad Prism software was used to identify the outliers and calculate statistical significance. The outliers were identified using the Robust Regression and Outlier Removal (ROUT) method and removed prior to the analysis of statistical differences. Statistical analysis of experiments with two experimental groups were assessed using unpaired student’s t-test. Experiments with more than two experimental groups were assessed using One-way ANOVA or two-way ANOVA with Tukey’s test. All data was presented as mean ± SD unless noted otherwise. Pearson correlative coefficient was used to conduct correlative analysis. All statistical details can be found in the figure legends.

## Supplementary Material

1

Supplemental information can be found online at https://doi.org/10.1016/j.celrep.2025.115831.

## Figures and Tables

**KEY RESOURCES TABLE:** 

REAGENT or RESOURCE	SOURCE	IDENTIFIER

Antibodies		

Rat monoclonal anti-CD31 (PECAM1)	BD Pharmingen	Cat#550274; RRID: AB_393571
Mouse monoclonal anti-CD31 (PECAM1)	Abcam	Cat# ab9498; RRID: AB_307284
Goat polyclonal anti-Collagen IV	EMD Millipore	Cat#AB769; RRID: AB_92262
Goat polyclonal anti-Collagen IV	Bio-Rad	Cat# 134001; RRID: AB_2082646
Rabbit polyclonal anti-Claudin-5	Invitrogen	Cat#34–1600; RRID: AB_2533157
Rabbit polyclonal anti-Occludin	Invitrogen	Cat#71–1500; RRID: AB_2533977
Mouse monoclonal anti-Occludin	Invitrogen	Cat#33–1500; RRID: AB_2533101
Rabbit monoclonal anti-Occludin	Abcam	Cat# ab167161; RRID: AB_2756463
Mouse monoclonal anti-ZO-1	Invitrogen	Cat#33–9100; RRID: AB_2533147
Rabbit polyclonal anti-N-cadherin	Abcam	Cat# ab18203; RRID: AB_444317
Rabbit polyclonal anti-pAkt (S473)	Cell Signaling Technology	Cat#9271; RRID: AB_329825
Rabbit polyclonal anti-Akt	Cell Signaling Technology	Cat#9272; RRID: AB_329827
Rabbit monoclonal anti-PI3K p110α	Cell Signaling Technology	Cat#4249; RRID: AB_2165248
Rabbit monoclonal anti-PI3K p110β	Cell Signaling Technology	Cat#3011; RRID: AB_2165246
Rabbit monoclonal anti-PI3K p110δ	Cell Signaling Technology	Cat#34050; RRID: AB_2799043
Rabbit monoclonal anti-Akt1	Cell Signaling Technology	Cat#2938; RRID: AB_915788
Rabbit monoclonal anti-Akt2	Cell Signaling Technology	Cat#3063; RRID: AB_2225186
Mouse monoclonal anti-Akt3	Cell Signaling Technology	Cat#8018; RRID: AB_10859371
Rabbit polyclonal anti-pAkt3	Invitrogen	Cat# PA5-12898; RRID: AB_10981731
Rabbit polyclonal anti-CTNNB1 (β-catenin)	Sigma-Aldrich	Cat#SAB4300470; RRID: AB_10634620
Rabbit polyclonal anti-p120 catenin	Santa Cruz	Cat#sc-1101; RRID: AB_632091
Mouse monoclonal anti-p120 catenin	Santa Cruz	Cat#sc-23873; RRID: AB_2086394
Rat monoclonal anti-CD144 (VE-cadherin)	BD Pharmingen	Cat#555289; RRID: AB_395707
Goat polyclonal anti-VE-cadherin	Santa Cruz	Cat#sc-6458; RRID: AB_2077955
Rabbit polyclonal anti-SV2A	Abcam	Cat#ab32942; RRID: AB_778192
Mouse monoclonal anti-GAD67	Abcam	Cat#ab26116; RRID: AB_448990
Mouse monoclonal anti-β-actin	Santa Cruz	Cat#sc-47778; RRID: AB_626632

Bacterial and virus strains		

Dendra2-occludin adenovirus	This paper	N/A

Biological samples		

Human brain tissue	University of Illinois NeuroRepository	https://chicago.medicine.uic.edu/neurology-rehabilitation/neuro-research/university-of-illinois-neurorepository-uinr/

Chemicals, peptides, and recombinant proteins		

DAPI	Invitrogen	D1306
25% Glutaraldehyde	Electron Microscopy Sciences	16220
10% PFA	Electron Microscopy Sciences	15712
CaCl2 dihydrate	Electron Microscopy Sciences	12340
Acetate Buffer	Electron Microscopy Sciences	11482–56
Osmium Tetraoxide	Electron Microscopy Sciences	19150
0.1 N Hydrochloric acid	Electron Microscopy Sciences	16760
Uranyl Magnesium Acetate	Electron Microscopy Sciences	22500
EMbed-812/DER 73 (EPON) kit	Electron Microscopy Sciences	14130
0.2 M Sodium cacodylate	Electron Microscopy Sciences	11652
Ethanol	Electron Microscopy Sciences	15055
Propylene oxide	Electron Microscopy Sciences	20412
Water Deionized, Reagent Grade A.C.S.	Electron Microscopy Sciences	22800–01
(1-ethyl-3-(3-dimethylaminopropyl) carbodiimide hydrochloride) (EDC)	ThermoFischer	22980
EDTA	Research Products International	E57020-500
Sodium Citrate Dihydrate	Fisher Chemical	S279-500
Polysorbate 20 (Tween 20)	Fisher BioReagents	BP337-100
Imidazole	Sigma Aldrich	I5513
Sodium Carbonate	Fisher CHemical	S263-500
*N*-(5-Amino-1-carboxypentyl)iminodiacetic acid (AB-NTA free acid)	Dojindo Molecular Technologies	A459-10
N-Hydroxysuccinimide (NHS)	Sigma Aldrich	130672
(3-Glycidyloxypropyl)trimethoxysilane (Glymo)	Sigma Aldrich	440167
N-cadherin Protein (His tag)	Sino Biological	11039-H08H
Ethanol	Decon Labs	04-355-224
Acetone	Fisher Chemical	A18-4
12 N Hydrochloric Acid	Fisher Chemical	A144S-500
5 N Sodium Hydroxide	Fisher Chemical	SS256-500
Dulbecco’s Phosphate-Buffered Saline, 1 X with calcium and magnesium	Corning	21-030-CM
Triton X-100	Fisher BioReagents	BP151
Collagen	MilliporeSigma	C8919
Fibronectin	MilliporeSigma	F1141
Tamoxifen	Sigma Aldrich	T5648
Corn Oil	Sigma Aldrich	C8267
OCT Compound	Fischer Scientific	23-730-571
Complete Classic Medium With Serum and CultureBoost^™^	Cell Systems	4Z0-500
Fluoromount G	SouthernBiotech	0100–01
Bovine Serum Albumin (BSA)	Sigma Aldrich	A7906
4x Laemmli Sample Buffer	Bio-Rad Laboratories	1610747
RIPA Buffer	Sigma Aldrich	R0278
Sodium Fluoride	Fischer Scientific	S299-100
HEPES	Gibco	15630080
10% sodium dodecyl sulfate (SDS)	Bio-Rad Laboratories	1610416
Sodium orthovanadate	Milipore Sigma	450243-10G
Protease Inhibitor Cocktail	Sigma Aldrich	P8340
Phosphatase Inhibitor Cocktail 2	Sigma Aldrich	P5726
Phosphatase Inhibitor Cocktail 3	Sigma Aldrich	P0044
Pierce ECL Western Blotting Substrate	Thermo Scientific	PI32106
Western Blot Stripping Buffer	Thermo Scientific	PI46430
Basic Nucleofector Kit for Primary Mammalian Endothelial Cells	Amaxa	VPI-1001
GeneSilencer	Genlantis	T500750
Wortmannin	TOCRIS Bioscience	CAS# 19545-26-7
Copanlisib	Selleckchem	S2802

Critical commercial assays		

Pierce BCA Protein Assay Kit	Thermo Scientific	PI23225

Experimental models: Cell lines		

Primary Human Brain Microvascular Endothelial Cells	Cell Systems	ACBRI 376

Experimental models: Organisms/strains		

*Cdh2* flox/flox (fL/fL)	Jackson	Jackson: B6.129S6(SJL)-Cdh2tm1Glr/J
*Cdh2* fl/fl iEC-KO	Kruse et al.^51^	N/A

Oligonucleotides		

Silencer Negative Control No. 1 siRNA (Scramble control)	Invitrogen	AM4635
ON-TARGETplus Human PIK3CA SMARTpool siRNA: GCGAAAUUCUCACACUAUU GUGGUAAAGUUCCCAGAUA GCUUAGAGUUGGAGUUUGA GACCCUAGCCUUAGAUAAA	Horizon Discovery	L-003018-00-0005
ON-TARGETplus Human PIK3CB SMARTpool siRNA: GGAUUCAGUUGGAGUGAUU GGCGGUGGAUUCACAGAUA GAUUAUGUGUUGCAAGUCA CCAUAGAGGCUGCCAUAAA	Horizon Discovery	L-003019-00-0005
ON-TARGETplus Human PIK3CD SMARTpool siRNA: ACGAUGAGCUGUUCCAAGUA CCAAAGACAACAGGCAGUA GCGUGGGCAUCAUCUUUAA CGAGUGAAGUUUAACGAAG	Horizon Discovery	L-006775-00-0005
ON-TARGETplus Human CDH2 SMARTpool siRNA: GUGCAACAGUAUACGUUAA GGACCCAGAUCGAUAUAUG CAUAGUAGCUAAUCUAACU GACAGCCUCUUCUCAAUGU	Horizon Discovery	L-011605-00-0005
ON-TARGETplus Human Akt1 (207) SMARTpool siRNA: CAUCACACCACCUGACCAA ACAAGGACGGGCACAUUAA CAAGGGCACUUUCGGCAAG UCACAGCCCUGAAGUACUC	Horizon Discovery	L-003000-00-0005
ON-TARGETplus Human Akt2 (208) SMARTpool siRNA: ACACAAGGUACUUCGAUGA GCAAGGCACGGGCUAAAGU GUGAAUACAUCAAGACCUG CAUGAAUGACUUCGACUAU	Horizon Discovery	L-003001-00-0005
ON-TARGETplus Human Akt3 (10000) SMARTpool siRNA: GCACACACUCUAACUGAAA GAAGAGGGGAGAAUAUAUA GUACCGUGAUCUCAAGUUG GACAGAUGGCUCAUUCAUA	Horizon Discovery	L-003002-00-0005

Recombinant DNA		

PH-Akt-mVenus	Klomp et al.^70^	N/A
CMV-Dendra2-Occludin	This paper	N/A
pShuttle-CMV-Dendra2-Occludin	This paper	N/A
mEmerald-Occludin-C-14	Addgene	54211
GFP-occludin S471D	Bolinger et al.^79^	N/A
GFP-occludin S471A	Bolinger et al.^79^	N/A
Dendra2-occludin S471D	This paper	N/A
Dendra2-occludin S471A	This paper	N/A

Software and algorithms		

ImageJ (FIJI)	National Institute of Health	https://fiji.sc/
Prism 10	GraphPad	https://www.graphpad.com/scientific-software/prism/
ANY-maze	Stoelting Co.	https://www.any-maze.com/

Other		

High performance No 1.5 Coverslip	Zeiss	474030-9000-000

## Highlights

- N-cadherin contacts and occludin TJs are destabilized in brain capillaries during aging
- Genetic deletion of N-cadherin in endothelial cells destabilizes occludin TJs
- N-cadherin mutant mice exhibit deficits in short-term memory
- N-cadherin stabilizes occludin at TJs via the PI3Kβ-Akt3 pathway
