# Supplementary material for: Deficiency in N-cadherin-Akt3 signaling impairs the blood-brain barrier
Source: Cell Rep. Author manuscript; Available in PMC 2025 Jul 13. (PMC12255523; doi:10.1016/j.celrep.2025.115831)
Supplement: 1 [file NIHMS2092693-supplement-1.pdf]

Cell Reports, Volume *44*

Supplemental information

Deficiency in N-cadherin-Akt3 signaling impairs the blood-brain barrier

Quinn Lee, Wan Ching Chan, Shuangping Zhao, Harry M. Hailemeskel, Riya Thomas, Mohsin Zafar, Fozia Mir, Peter T. Toth, Kamran Avanaki, Leon M. Tai, Jeffrey Loeb, and Yulia A. Komarova

Figure S1. Endothelial N-cadherin stabilizes VE-cadherin, occludin, and ZO-1 at inter-endothelial junctions, related to Figure 1

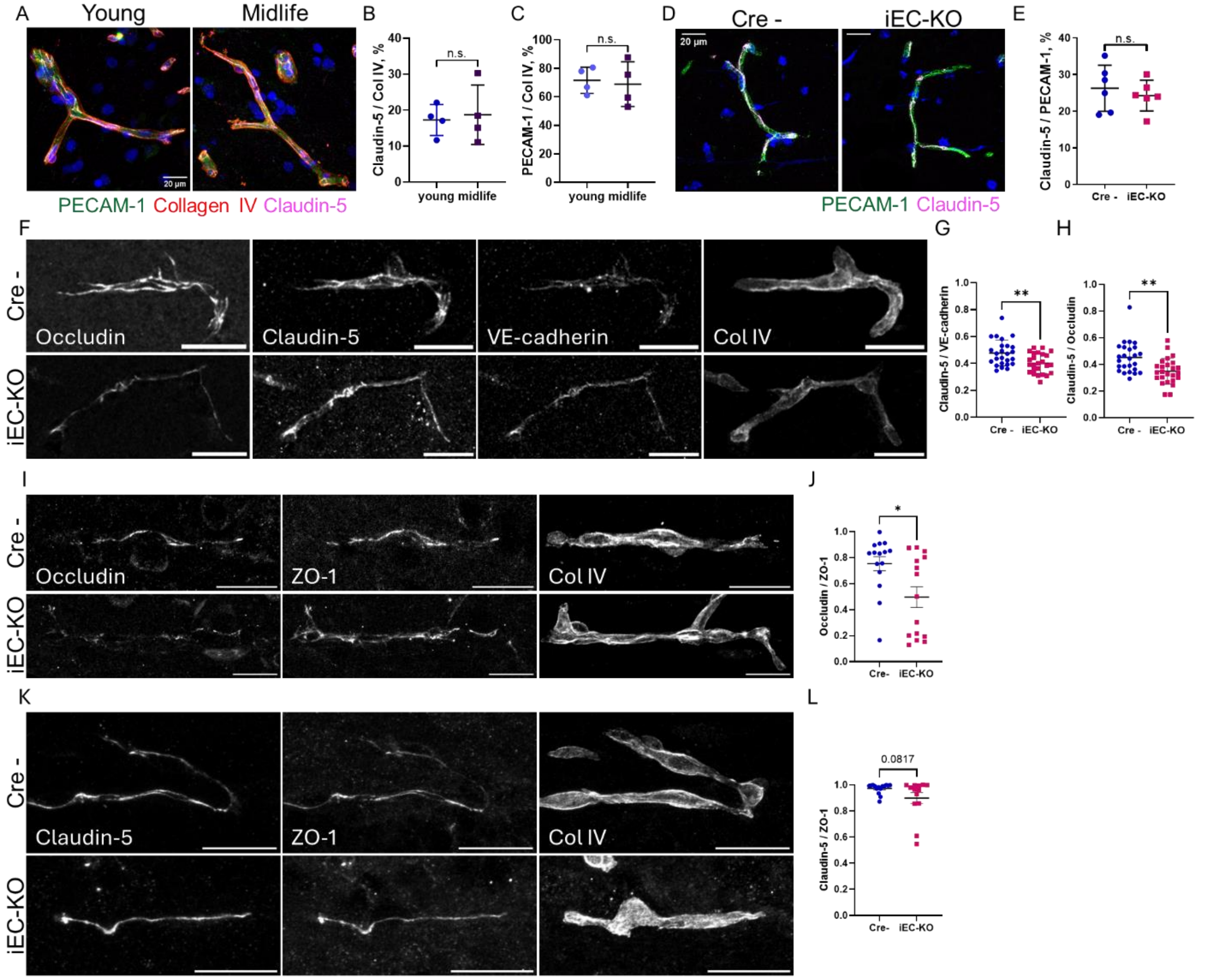

A) Immunofluorescent staining for PECAM-1 (green), collagen IV (red), claudin-5 (magenta), and DAPI (blue) of cortex tissues from young and middle-aged individuals. Scale bar, 20 µm. **B-C)** Quantification of claudin-5 (B) and PECAM-1 (C) junctional areas normalized to collagen IV. n=4 human samples per group; mean ± SD. n.s., not significant by two-tailed, unpaired *t*-test. **D)** Immunofluorescent staining of mouse cortex tissues for claudin-5 (magenta) and PECAM-1 (green) from N-cadherin flox/flox (control) and iEC-KO mice. Scale bar, 20 µm **E)** Quantification of claudin-5 junctional area normalized to PECAM-1. n=6 mice per group; mean ± SD. n.s., not significant by two-tailed, unpaired *t*-test. **F)** Correlative immunofluorescent analysis of VE-cadherin adherens junctions, as well as occludin and claudin-5 tight junctions, within the same vessels in the hippocampus of control (Cre-) and *Cdh2* iEC-KO mice. Scale bar, 20 µm. **G-H)** Mander's co-efficient for claudin-5 and occludin **(G)** and claudin-5 and VE-cadherin **(H)**. n=25 fields per group in 5 mice; mean ± SD. **, p<.01 by two-tailed, unpaired *t*-test. **I-L**) Co-localization immunofluorescence analyses of occludin and ZO-1 **(I)** or claudin-5 and ZO-1 (**K**) within different sets of vessels in the hippocampus of control (Cre-) and *Cdh2* iEC-KO mice. Scale bar, 20 µm. **J, L)** Mander's co-efficient for occludin and ZO-1 **(G)** and claudin-5 and ZO-1 **(K)**. n=5-10 fields per group in 3 mice; mean ± SD. **, p<.01 by two-tailed, unpaired *t*-test.

Figure S2. Endothelial N-cadherin does not alter expression of junctional proteins, related to Figure 1

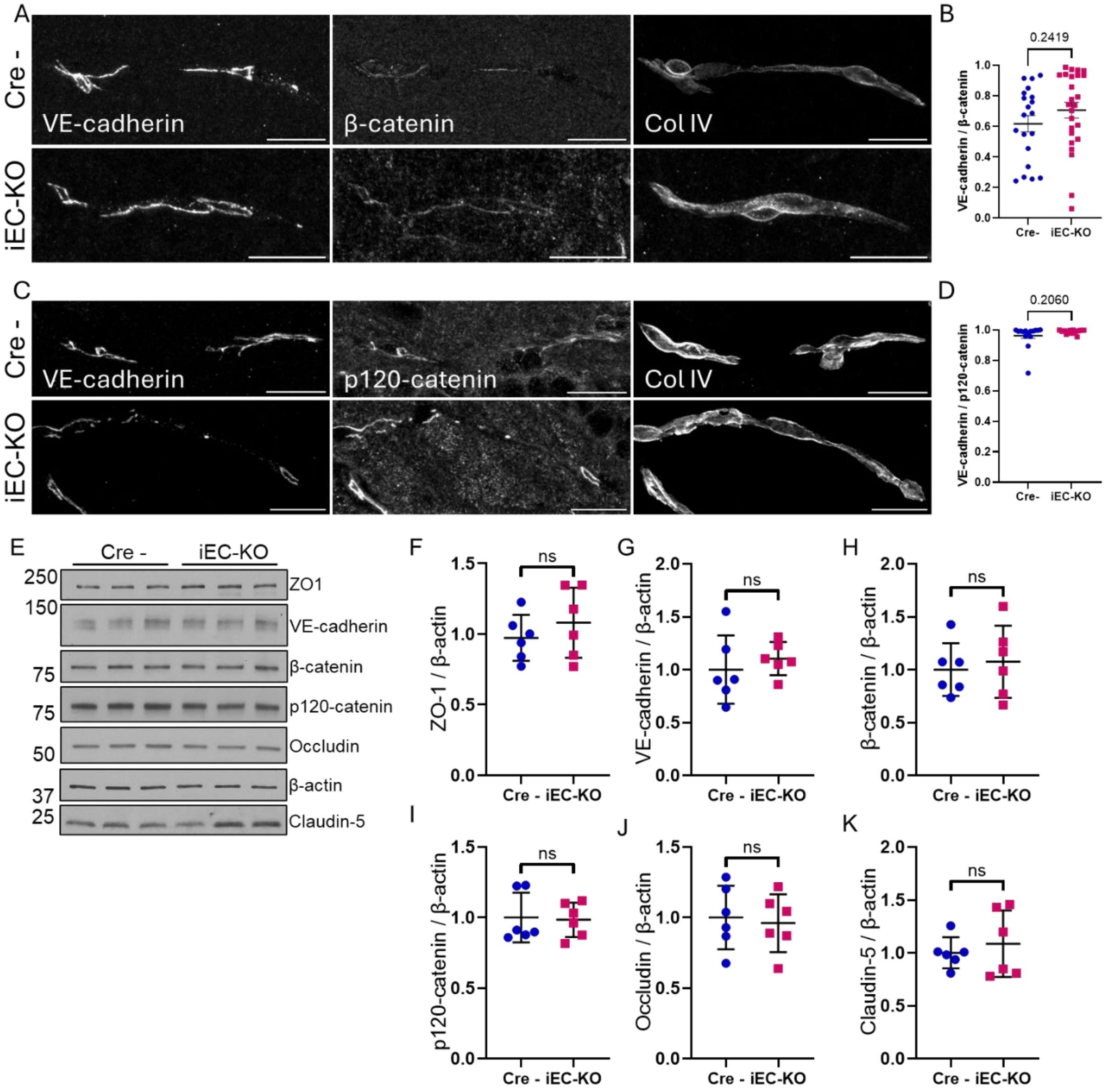

A-D) Co-localization immunofluorescence analyses of VE-cadherin and β-catenin **(A-B)** or VE-cadherin and p120-catenin **(C-D)** within different sets of vessels in the hippocampus of control (Cre-) and *Cdh2* iEC-KO mice. Scale bar, 20 μm. **B,D)** Mander's co-efficient for VE-cadherin and β-catenin (B) or VE-cadherin and p120 (D). n=5-10 fields per group in 3 mice; mean ± SD. *, $p<0.05$ and **, $p<.01$ by two-tailed, unpaired *t*-test. **E)** Western blot analysis of BEC junctional proteins, ZO1, VE-cadherin, β-catenin, p120-catenin, occludin, claudin-5, and β-actin in the cortex tissue of control and iEC-KO mice. **F-K)** Quantification of the relative expression of ZO1 **(F)**, VE-cadherin **(G)**, β-catenin **(H)**, p120-catenin **(I)**, occludin **(J)**, and claudin-5 **(K)** in **E**. n = 6 mice per group; mean ± SD. n.s., not significant by two-tailed, unpaired *t*-test.

Figure S3. Endothelial-pericyte N-cadherin contacts support spatial memory, related to Figure 2

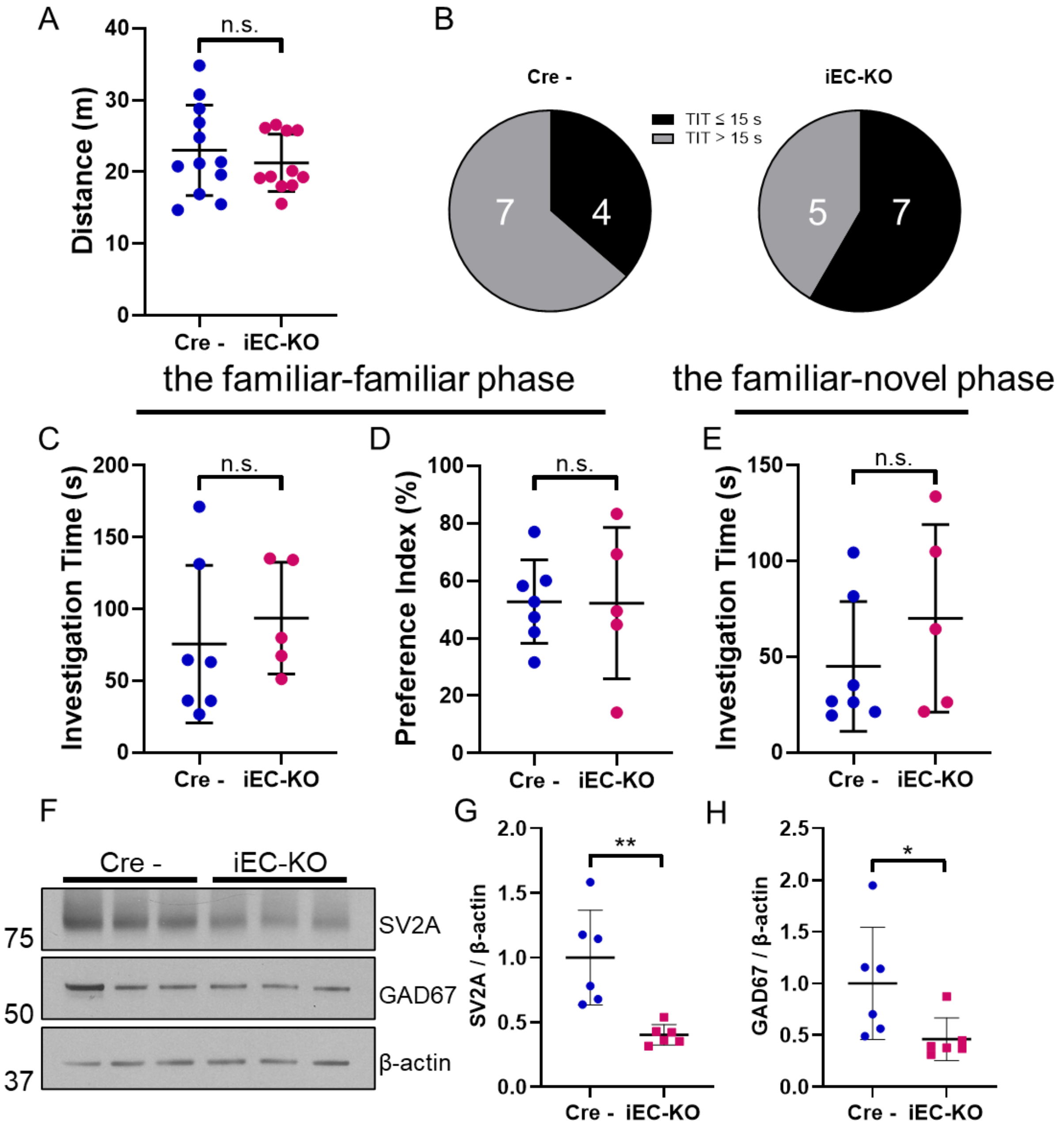

A) The distance traveled by control and *Cdh2* iEC-KO mice during open field behavioral test. n=11-12 mice per group. n.s., not significant by two-tailed, unpaired *t*-test. **B-E)** Analyses of investigation time exclusion criteria in novel object recognition test **(B)**. Mice with a total investigation time ≤ 15 seconds (black) in either familiar-familiar or familiar-novel phase were excluded. n=11-12 mice per group**.** Total investigation time **(C)** and preference index **(D)** during the familiar-familiar phase; and the total investigation time during the familiar-novel phase **(E)** of novel object recognition test. n=5-7 mice per group; mean ± SD. n.s., not significant by two-tailed, unpaired *t*-test. **F)** Western blot analysis of SV2A, GAD67, and β-actin in the hippocampus tissue of control and iEC-KO mice. **G-H)** Quantification of the relative expression of SV2A **(G)** and GAD67 **(H)** in **F**. n = 6 mice per group; mean ± SD. *, p<.05; **, p<.01 by two-tailed, unpaired *t*-test.

Figure S4. N-cadherin contacts activate PI3K-Akt signaling, related to Figures 3 and 4

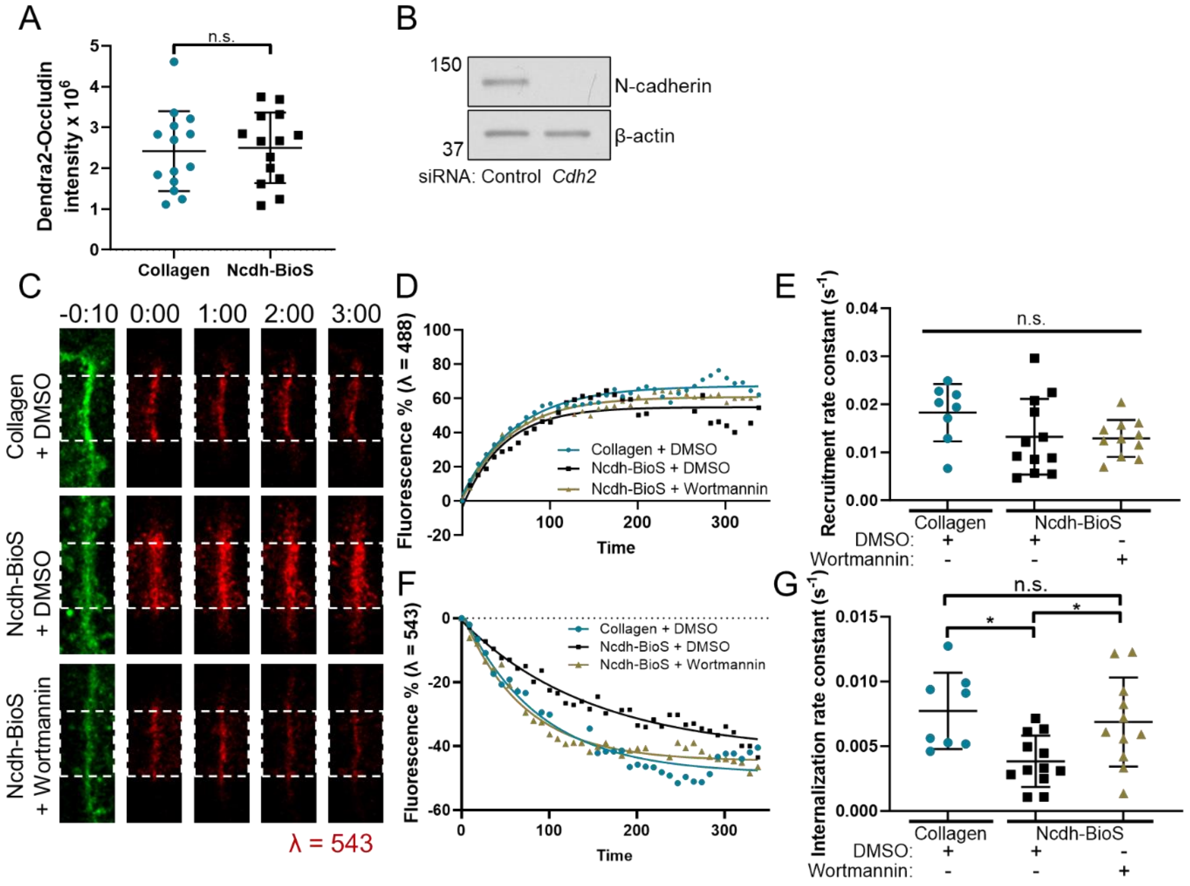

A) Dendra2-occludin expression in BECs grown on either collagen or Ncdh-BioS assessed as fluorescent intensity. n=14 cells per condition. n.s., not significant by two-tailed, unpaired *t*-test. **B)** Western blot analysis of N-cadherin and β-actin in BECs after treatment with control or *Cdh2* siRNA. **C)** Timelapse images of Dendra2-occludin at TJs as in **Fig. 3E** before and after Dendra-2 photoconversion in BECs grown on either collagen or Ncdh-BioS and treated with DMSO (control) or 100 nM PI3K inhibitor wortmannin. **D** and **F)** Representative graphs of occludin recruitment **(D)** and internalization **(F)** rates at TJs. **E** and **G)** Recruitment **(E)** and internalization **(G)** rate constants (*k*) in **D, F**. n=8-12 junctions; mean ± SD. *, p<.05 by ANOVA with Tukey's post hoc test.

Figure S5. N-cadherin contacts stabilize occludin at TJs via PI3K signaling, related to Figures 4 and 5

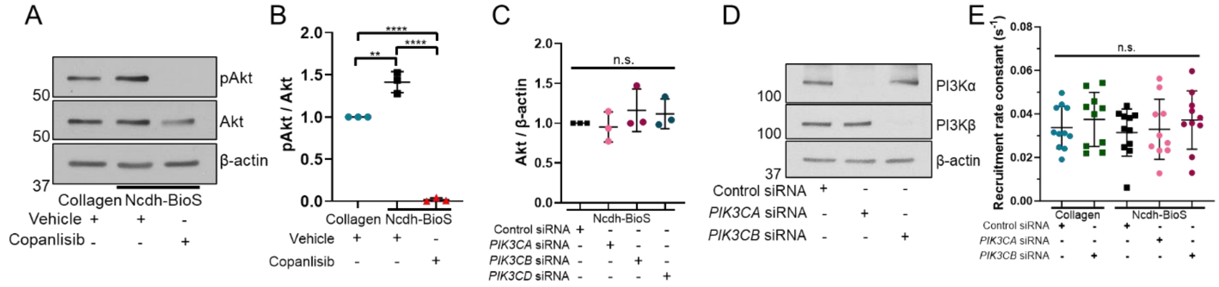

A) Western blot analyses of occludin, pAkt, Akt, and β-actin in BECs grown on either collagen or Ncdh-BioS and treated with vehicle or 100 nM copanlisib. **B)** Quantification of relative pAkt to Akt ratio in **A**. n= 3 experiments; mean ± SD. **, p<.01; ****, p<.0001 by ANOVA with Tukey's post hoc test. **C)** Quantification of relative expression of total Akt in **Fig. 4E**. n = 3 experiments; mean ± SD. n.s., not significant. **D)** Western blot analyses of PI3Kα, PI3Kβ and β-actin protein expression in BECs after treatment with control, *PIK3CA*, or *PIK3CB* siRNA. **E)** Recruitment rate constants of Dendra2-occludin at TJs in BECs monolayers grown on Ncdh-BioS and treated with control, *PIK3CA*, or *PIK3CB* siRNA. n=10-11 junctions, mean ± SD. n.s., not significant by ANOVA with Tukey's post hoc test.

Figure S6. N-cadherin contacts stabilize occludin at TJs via Akt3 signaling, related to Figures 6 and 7

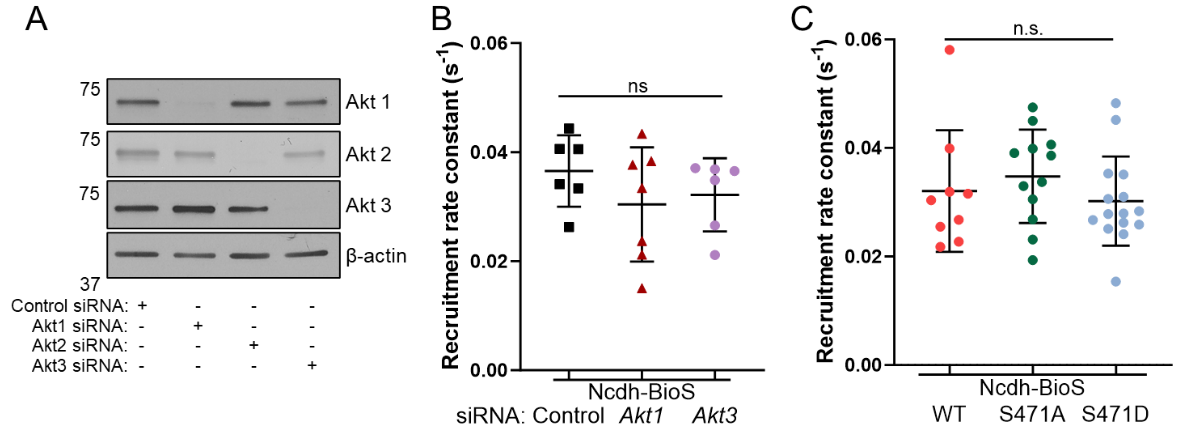

A) Western blot analyses of protein expression of Akt1, Akt2, Akt3, and β-actin in BECs after treatment with control, *Akt1, Akt2*, or *Akt3* siRNA. **B)** Recruitment rate constants of Dendra2-occludin to TJs in BECs monolayers grown on Ncdh-BioS and treated with control, *Akt1*, or *Akt3* siRNA. n=6-7 junctions; mean ± SD. n.s., not significant by ANOVA with Tukey's post hoc test. **C)** Recruitment rate constant of Dendra2-occludin wildtype, S471D or S471A mutants at TJs in BECs monolayers grown on either collagen or Ncdh-BioS. n=9-15 junctions; mean ± SD. n.s., not significant by ANOVA with Tukey's post hoc test.
